# Effectiveness of Serious Games as Digital Therapeutics for Enhancing the Abilities of Children With Attention-Deficit/Hyperactivity Disorder (ADHD): Systematic Literature Review

**DOI:** 10.2196/60937

**Published:** 2025-05-06

**Authors:** Jing Lin, Woo-Rin Chang

**Affiliations:** 1 Department of Digital Contents College of Art and Design Kyung Hee University Yongin-si Republic of Korea

**Keywords:** serious games, ADHD, attention deficit disorder with hyperactivity, neurodevelopmental disorders, digital therapeutics, DTx, systematic review, pediatric, children

## Abstract

**Background:**

Attention-deficit/hyperactivity disorder (ADHD) is a neurodevelopmental disorder that often begins in childhood and requires long-term treatment and management. Given the potential adverse effects of pharmacological interventions in children, interest in alternative treatments has increased. Among alternative therapies, serious games have emerged as a promising digital therapeutic approach and are increasingly recognized as an important intervention for children with ADHD.

**Objective:**

This systematic review aims to evaluate the effectiveness of serious games as digital therapeutics for children with ADHD. It focuses on assessing therapeutic outcomes, including improvements in attention, hyperactivity-impulsivity, social skills, motor skills, executive functions, and enjoyment.

**Methods:**

The review was conducted following PRISMA (Preferred Reporting Items for Systematic Reviews and Meta-Analyses) guidelines. A comprehensive literature search was performed across 5 databases: PubMed, Web of Science, Scopus, IEEE Xplore, and ACM Digital Library, covering English studies published from January 2010 to January 2024. Eligibility criteria were established based on the PICOS (Participants, Intervention, Comparison, Outcomes, Study design) framework, with digital therapeutics guidelines pragmatically applied to inform inclusion criteria, exclusion criteria, and quality assessment. Standardized tools including the Cochrane Risk of Bias Tool for randomized controlled trials, the Cochrane Risk of Bias Tool for Non-Randomized Studies of Interventions (ROBINS-I) for nonrandomized controlled trial studies, and the Critical Appraisal Skills Program checklists were used to evaluate risk of bias. Data on study design, targeted abilities, game software and hardware, and intervention parameters (duration, frequency, and length) were extracted and synthesized descriptively.

**Results:**

Of the 35 studies identified (1408 participants), gender data were available for 22 studies (888 participants), comprising 660 male and 228 female participants. Analysis revealed multiple abilities focused across many studies: 80% (28/35) assessed attention, 29% (10/35) addressed hyperactivity-impulsivity, 17% (6/35) explored improvements in social skills, 20% (7/35) evaluated motor skills, and 43% (15/35) investigated executive functions. Furthermore, in 89% (31/35) of the trials, children exhibited a positive attitude toward game interventions. Evidence suggests that serious games may contribute to improvements in attention, hyperactivity-impulsivity, social skills, and executive functions in children with ADHD. Although findings on motor skills were inconclusive, interventions incorporating somatosensory inputs demonstrated benefits for hand-eye coordination.

**Conclusions:**

The findings support the potential of serious games as a digital therapeutic modality for children with ADHD, offering benefits in core symptoms and associated impairments while promoting engagement.

**Trial Registration:**

PROSPERO CRD420250509693; https://www.crd.york.ac.uk/PROSPERO/view/CRD420250509693

## Introduction

### Background

Attention-deficit/hyperactivity disorder (ADHD) is a neurodevelopmental disorder [1], often diagnosed in childhood and persisting into adolescence and adulthood [2]. It is associated with a high rate of comorbidity, accidents, and mortality, among others [3,4]. Although ADHD medications exhibit significant therapeutic effects [5], some guardians are reluctant to medicate their children due to potential long-term adverse developmental effects and varying effectiveness across different ADHD subtypes [6,7]. Furthermore, the core symptoms of ADHD, which include inattention and hyperactivity-impulsivity, typically necessitate multiple therapeutic interventions to ensure effective management [8].

Serious games, which have emerged as a promising alternative therapy, are defined as “games that do not have entertainment, enjoyment or fun as their primary purpose” [9]. Serious games are used in medical diagnostics, therapy, prevention, health promotion, and medical or patient education [10]. Serious games have been proposed for neurodevelopmental disorder interventions primarily due to their appeal, engagement, and effectiveness [11,12]. Meanwhile, music therapy [13], exercise therapy [14], and chess therapy [15,16], shown to be effective for managing ADHD symptoms in children, can achieve better therapeutic effects when combined with serious games.

In 2020, EndeavorRx became the first US Food and Drug Administration–authorized prescription digital therapeutic (DTx) specifically designed for pediatric ADHD, leveraging a video game experience to enhance attention in children with this condition [17]. Subsequently, in June 2023, the International Organization for Standardization (ISO) introduced ISO/TR 11147, which formally defined “Digital Therapeutics” as evidence-based, software-driven interventions to prevent, manage, or treat a medical disorder or disease [18]. This standardization by ISO facilitates industry and global alignment regarding the scope and implementation of DTx.

To date, only 2 systematic reviews have specifically examined the effects of games on children with ADHD [19,20], revealing the potential of such interventions to address core ADHD symptoms and underscoring the need for greater collaboration between developers and health care professionals. However, children with ADHD manifest a range of symptoms beyond the core symptoms of inattention and hyperactivity-impulsivity [21], including challenges in social interaction, impaired motor skills, and deficits in executive functioning [22,23]. In addition, further research on nonpharmacological treatments targeting specific abilities in pediatric patients with ADHD indicates that interventions designed to enhance social, motor, and executive functioning skills may potentially benefit overall ADHD symptom management [24]. The role of environmental and familial factors, such as maternal depression and emotional attitudes, has also been convincingly demonstrated to impact the development of executive functions in children [25]. Consequently, an UpToDate literature review is imperative to address existing gaps in research on capability enhancement, and examining player enjoyment remains critical.

The current evidence is insufficient to demonstrate the specific impact of serious games on children with ADHD, mainly because of several limitations: the limited number of studies included, the insufficient variety of games, and the missing and inconsistent reporting of results. Therefore, this review aimed to include more appropriate studies, expand the coverage of serious games, and diversify the outcome assessments, so that a more comprehensive review could be conducted to further systematically assess the available evidence related to the impact of serious games on the various abilities of children with ADHD.

### Objectives

Compared with other systematic reviews, this study offers 3 unique contributions: First, it provides a comprehensive review of the most recent studies over the past 14 years, using the basic definition of DTx as part of the inclusion and exclusion criteria, thereby addressing the gap in recent studies that adhere to industry standards. Second, it offers a broader evaluation of the impact of serious games as interventions on children’s competencies, including attention, hyperactivity-impulsivity, social skills, motor skills, and executive functions. Third, it discusses outcomes related to participation, like enjoyment and adverse effects.

## Methods

### Overview

A total of 2 literature search phases were conducted in January 2024 and February 2025. The exploratory search phase identified 4420 records. Using Zotero Reference Management software, we automatically removed 1022 duplicate records. Subsequently, we manually deleted an additional 7 duplicates and 1 retracted article, resulting in a final set of 3390 records. Based on the exploration, the second search yielded 5109 records. After removing 1492 duplicate records and 2 retracted articles, and cross-validating with the exploratory search, 5510 records were retained for evaluation. The PICOS framework (Participants, Intervention, Comparison, Outcomes, Study design) informed both search strategy development and eligibility criteria formulation. This systematic review adhered to the PRISMA (Preferred Reporting Items for Systematic Reviews and Meta-Analyses) guidelines (Multimedia Appendix 1) [26]. In addition, we followed the recommendations of the Cochrane Consortium for conducting systematic reviews and used the RefHunter website for guidance [27]. For the final selection of 35 articles, established assessment tools were used, including the Cochrane Risk of Bias Tool for randomized controlled trials (RCTs), the Cochrane Risk of Bias Tool for Non-Randomized Studies of Interventions (ROBINS-I), and the CASP (Critical Appraisal Skills Programme) checklists to evaluate risk of bias of each study.

### Identification: Literature Search

The PICOS criterion [28] was used to guide the literature search strategy and identify inclusion and exclusion criteria. During the development of the literature search strategy, to ensure that the search covered a wider range of related studies, the search strategy was based on the broader P (Participants, patients with ADHD) and I (Intervention, games) without strictly limiting C (Comparison), O (Outcomes) and S (Study design). This is because existing studies cover a wide range of experimental designs, including exploratory and qualitative studies with no control group. A strict search strategy may limit the scope of studies, resulting in potentially valuable literature being missed. Also, the search strategy did not limit the age, but we limited the inclusion criteria to children with ADHD. Considering the interdisciplinary nature of this review, the selected databases should include relevant research literature from diverse disciplines, extending beyond psychology, game design, computer science, and medicine. Consequently, databases such as PubMed, Web of Science, Scopus, IEEE Xplore, and ACM Digital Library were chosen to address the needs of interdisciplinary research.

Close synonyms for “Serious Games” include “gamification,” “gamified,” “game mechanics,” “game dynamics,” “game design,” “game based,” and “gaming.” [29]. To minimize limitations, “DTx” and “DHI” (digital health interventions) were added to the search terms. DTx is considered a subset of “DHI,” a broader term that encompasses the use of digital technologies to support wellness and health care practices, particularly in the management of neurodevelopmental disorders [30-32].

A 2-phase literature search strategy was used. The initial exploration phase spanned January to March 2024. Relevant search terms for “ADHD” and “Serious Games” were identified by systematically mapping domain-specific subject headings and keywords informed by expert knowledge. The exploratory phase identified 4420 publications, with deduplication yielding 3390 records. Index terms, titles, and abstracts of retrieved articles were systematically analyzed to extract search terms informing subsequent query optimization. Relevant articles from the exploratory phase were archived for cross-verification during the second search screening.

The second search implementation occurred in February 2025, incorporating emergent terminology from exploratory phase findings, including educational gaming, exergaming, cognitive training, telemedicine, and mobile health. Consultations with a medical research librarian and pediatric ADHD specialist informed MeSH (Medical Subject Headings) term selection and Boolean operator construction (Multimedia Appendix 2).

### Screening: Title and Abstract Screening

PICOS criterion guided inclusion and exclusion criteria and added other related labels. Duplicates were automatically identified using Zotero software (version 6.0.29, Corporation for Digital Scholarship, Roy Rosenzweig Center for History and New Media). Following iterative deduplication, publications underwent manual screening. Initially, 2 authors independently screened the titles and abstracts of relevant studies. The full texts of eligible studies were subsequently assessed independently by the same authors using inclusion criteria, with any arising conflicts resolved through conference discussions. Each study was evaluated against these criteria, resulting in 5210 exclusions for various reasons, including inappropriate target population, interventions not involving a game, unreasonable experimental design, nonjournal publication, and language not being English (Table 1).

**Table 1:** Title and abstract screening Participants, Intervention, Comparison, Outcomes, Study design (PICOS) diagram for children with attention-deficit/hyperactivity disorder (ADHD) game interventions (2010-2024). The systematic review screening process for ADHD children game studies is based on the PICOS framework.

	Inclusion criteria	Exclusion criteria^a^
Patient	Children diagnosed with ADHD.	Studies that did not only include children with ADHD and/or their caregivers (guardians, teachers, and clinicians). Nonhuman studies.
Intervention	Research focused on serious games designed specifically for targeting ADHD: cognitive training games, neurofeedback-based games, exergames, and social skills training games.	Studies focused on other interventions (eg, pharmacological interventions, Pele medicine, mobile health and sensors, web-based intervention, augmented or virtual reality, and robot assistants).
Comparison	The comparative arms could include various interventions or usual care.	—^b^
Outcome	Focus on outcomes in the following developmental domains: attention, hyperactivity-impulsivity, social skills, motor skills or physical activities, executive functions, enjoyment, and intervention adherence.	Studies lacking concrete examples or evidence. Examples are not games or game-based platforms or software. Studies where the original text cannot be retrieved. Repeated trials.
Study Design	Any experimental or quasi-experimental evaluative design, including pilot and feasibility studies. Nonrandomized studies (eg, pre-post study with no control group). Cohort or longitudinal studies that had pre-post outcome measures. Case series or case studies.	Theoretical designs or frameworks without example data. Studies addressing conditions other than ADHD (eg, autism spectrum disorder, anxiety disorders, bipolar disorder, and learning disorders). Studies not aimed at treating or alleviating ADHD symptoms.
Publication Type	Peer-reviewed article. Full paper proceedings.	Conference abstracts, study protocols, books, websites, reviews, theses or dissertations, short conference paper proceedings, posters, and demos.
Publication period	From 1 January 2010 to 1 January 2024	Before 2010, after 1 January 2024
Setting	Any country or region	Not applicable
Language	English	Any other language
Aggregate	Included (300)	Excluded (5210)

^a^Exclusion criteria include nonrelevant study populations, interventions lacking a game component, inappropriate study designs, nonjournal publications, and non–English-language articles.

^b^Not applicable.

### Eligibility: Content Screening

Following the preliminary screening based on inclusion criteria, 300 studies were analyzed using the full-text exclusion assessment for children with ADHD game interventions as a benchmark for further content screening. Each study was evaluated against these criteria, resulting in 261 exclusions for various reasons such as lack of relevance to ADHD-only populations, excluding studies not focused on ADHD treatment, nonchild populations, nongame platforms, or lacking empirical data (Table 2). Subsequently, a quality assessment was conducted, where each criterion was rated as “yes,” “partially,” or “no,” and scored, respectively, with values of 1, 0.5, and 0. The final score, requiring a minimum of 4 out of 7 (about 60%) marks, was calculated as an average of the grades. The quality criteria and scoring methodology were based on research by Coelho et al [33]. This step led to excluding 4 articles that did not meet the minimum threshold of 0.6, while 35 other articles were included (Multimedia Appendix 3) [14,34-67].

In our literature screening process, it is crucial to ensure that the research selected develops a game specifically designed for treating children with ADHD, possesses a fundamental game structure, and is sufficiently comprehensive to allow for an analysis of its usefulness [68]. However, as most games evaluated are not yet commercially available, the 8 DTx guidelines were pragmatically applied to the design of the exclusion criteria and quality assessment, drawing upon the concept of the minimum viable product (MVP). Some guidelines were temporarily deprioritized (Table 3). Despite the limited availability of MVP-related content in digital contexts, the MVP concept continues to offer the potential for enhancing product development efficiency, quality, and innovation [69].

**Table 2:** Full-text exclusion assessment for children with attention-deficit/hyperactivity disorder (ADHD) game interventions. Details the full-text review phase, excluding studies not focused on attention deficit hyperactivity disorder treatment, nonchild populations, nongame platforms, or lacking empirical data.

Exclusion criteria		Excluded, n
**Full-text articles excluded**		
	Studies addressing conditions other than ADHD	62
	Studies that included not only children with ADHD and/or their caregivers	54
	Studies not aimed at treating or alleviating ADHD symptoms	41
	Studies lack concrete examples or evidence	35
	Examples are not games/game-based platforms or software	39
	Studies focused on other interventions	8
	Studies where the original text cannot be retrieved	4
	Repeated trials	18
**Quality assessment**		
	Substandard quality	4
**Aggregate**		
	Included: n=35	265

**Table 3:** Design of minimum viable product quality evaluation in children with attention-deficit/hyperactivity disorder game interventions as digital therapeutics. The 8 digital therapeutics guidelines were pragmatically integrated with minimum viable product principles to balance game design feasibility and therapeutic relevance.

DTx^a^ core principles	Reasons for deprioritization	Prioritization recovery
Incorporate design, manufacturing, and quality best practices.	—^b^	—
Engage end users in product development and usability processes.	—	—
Incorporate patient privacy and security protections.	—	—
Apply product deployment, management, and maintenance best practices.	During initial development and prototyping, the primary focus might be on validating the therapeutic concept and functionality rather than on the complexities of deployment and ongoing maintenance.	Applying product deployment, management, and maintenance becomes critical as the product approaches a state ready for broader testing or market launch, to ensure scalability, reliability, and user support.
Publish trial results inclusive of clinically meaningful outcomes in peer-reviewed journals.	—	—
Be reviewed and cleared or certified by regulatory bodies as required to support product claims of risk, efficacy, and intended use.	Early-stage products, particularly those in the research, discovery, or pre-clinical phases, may not yet have undergone regulatory review.	The process of obtaining regulatory clearance or certification often comes after proving the product's efficacy and safety, which are established through clinical trials.
Make claims appropriate to clinical evaluation and regulatory status.	In the exploratory stages of development, a DTx product might not yet have undergone extensive clinical evaluation, and thus cannot make definitive claims about its efficacy, safety, or intended use.	Formal claims become relevant and necessary as the product undergoes clinical trials and seeks regulatory approval.
Collect, analyze, and apply real-world evidence and/or product performance data.	—	—

^a^DTx: digital therapeutics.

^b^Not available.

### Included: Data Abstraction and Analysis

To screen the final record set for inclusion criteria, after manually removing duplicates (identical game instances with different paper titles), 2 authors used Zotero to further screen the publications manually. Together, the 2 authors determined keywords and selected databases in step 1. In the screening step, the first author screened the titles and abstracts of all records, applying inclusion criteria to identify obvious exclusions. Subsequently, in the eligibility step, the 2 authors independently reviewed the remaining papers and resolved any conflicts through discussion, arriving at the final decision.

Significant heterogeneity in study types, comparisons, intervention programs, and outcomes indicated that the data from this review were not suitable for meta-analysis. Therefore, descriptive analysis was used to summarize the impact of serious games on the rehabilitation of children with ADHD. The process of conducting descriptive analysis involved tabulating features of the original studies from 2 perspectives: experimental design and game design. This included software, description of gameplay, duration, length and frequency, sample size and characteristics, and sex of children participants. Jing Lin and Woo-Rin Chang independently extracted this information using predefined Microsoft Excel (Table 4).

Subsequently, the authors thoroughly reviewed and validated the collected data to ensure accuracy and reliability. In cases of disagreement, consensus was reached through discussion. Finally, the data were organized and categorized for each specific outcome to enable a comprehensive assessment of the findings.

**Table 4:** Characteristics of included studies.

Studies	Software, sample size, and characteristics	Description and sex of the child participants	Duration, length, and frequency
Giannaraki et al [34]	ADDventurous Rhythmical Planet 4 children (including 2 with ADHD^a^) and 4 expert educators	Players use the tin drum to produce rhythms and collaborate in multiuser mode to achieve common goals. 3 male and 1 female	—^b^
Rodrigo-Yanguas et al [35]	The Secret Trail of Moon 37 children and adolescents	Including 5 VR^c^ minigames, each aimed at enhancing specific cognitive skills. 25 male and 12 female	Single sessions for 10-40 min
Aghdam and Alavi [36]	MIND PRO Working Memory Game 6 children with ADHD, ages 6 to 11 years	In the auditory section of the game, players identify and select boxes that produce distinct sounds from multiple options The visual section requires players to select boxes that contain matching images 4 male and 2 female	30 min per session, 12 sessions total
Batista et al [37]	Taboo! 184 children with ADHD, ages 7-14 years	The game uses an endless runner format where the player avoids obstacles and solves math problems Predominantly male	—
Wiguna et al [38]	Indonesian computer-based game prototype 10 children with drug-naïve ADHD, aged 7-12 years	A role-playing game features children acting as fruit car drivers, tasked with delivering color-specific fruits to corresponding houses 9 male and 1 female	30 min per session, 20 sessions total over 4 weeks
Ou et al [39]	Fishing Master/Fruit Train/Ocean Manager 3 children aged 8-12 years old diagnosed with ADHD	The games enhance hand-eye coordination and balance through activities like catching fish, picking fruit on a moving train, and feeding fish while dodging obstacles 1 male and 2 female	40 min per session, 3 times a week for 3 months
Capelo et al [40]	Multisensory Virtual Game 20 children aged 7-12 years, including both children with and without ADHD	In a multisensory game, children use the Leap Motion controller to match virtual objects to containers based on their color and shape Mixed (10 children with ADHD and including 4 female and 6 male; 10 children without ADHD and including 4 female and 6 male)	—
García-Baos et al [41]	RECOGNeyes 28 children aged 8-15 years diagnosed with ADHD	In the word recognition task, participants need to identify the correct words displayed among scrambled words at the screen's center 18 male and 10 female	30 min per session, 3 times per week for 3 weeks
Avila-Pesantez et al [42]	ATHYNOS 11 children diagnosed with ADHD, aged 7-10 years	AR game, activities in the ATHYNOS prototype include “Drag and Drop” and “Shapes,” focusing on improving hand-eye coordination 9 male and 2 female	20 min per session, 2 times per week during a month
Kanellos et al [43]	REEFOCUS 75 children diagnosed with ADHD aged 8-14 years	A management simulation game set in an underwater environment where players engage in tasks	45 min per session for 8 weeks
Kollins et al [44]	AKL-T01(An early version of EndeavorRx) 206 children. 130 children continued stimulant medication during the study. 76 children, no ADHD medication.	Players control a character navigating a colorful, animated world on a fixed path 154 male and 52 female	12 weeks, split into two 4-week treatment phases with a 4-week pause in between Daily sessions of ~25 minutes, consisting of five 5-minute missions, 5 days a week
Machado et al [45]	3 children aged 7-12 years are diagnosed with ADHD	A dry a wireless electroencephalography headset captures the player’s brain activity, gameplay involves tasks like collecting stars or managing fuel levels 3 male	20-60 min per session
De la Guía et al [46]	StiCap 12 children with ADHD, ages 5-16 years	The games use tangible user interfaces with RFID technology for engaging learning activities 8 male and 4 female	—
Avila-Pesantez et al [47]	CIUDAD PUZZLE 20 children diagnosed with ADHD	A puzzle game that incorporates NFC tags to interact with physical game pieces 14 male and 6 female	15 min per session, 2 times per week for 3 months
Schena et al [48]	IAmHero 60 children with ADHD, age 5-12 years	The application features 3 VR mini-games to enhance various abilities 33 male and 27 female	30 min weekly for 6 months
Blandón et al [49]	Harvest Challenge 9 children diagnosed with ADHD, ages 5 to 12 years	Gameplay is controlled by monitoring attention levels via a Brain-Computer Interface (BCI)	30 min per session, 2 sessions total
Vita and Mennitto [50]	NEUROBOT A 10-year-old child diagnosed with ADHD	Participants control the speed of a Lego robot by concentrating and using brain wave activity to compete in races, aiming to increase the robot’s speed	30 min weekly
Chen et al [51]	10 children diagnosed with ADHD	The game features tasks that require players to respond in real time to changes in their brainwave status	Twice a week, 30-60 min per session for 14 sessions
Soysal et al [52]	Tetris 6 children, including 2 diagnosed with ADHD	The study explored how different types of background music affect the attention levels of children while playing Tetris	—
Weerdmeester et al [53]	Adventurous Dreaming Highflying Dragon 73 school-aged children with elevated ADHD symptoms	Players control avatars through full-body movements to solve cognitively challenging tasks in the game 58 male and 15 female	15 min per session, 2 times per week for 3 weeks
Jácome et al [54]	DIVIDI2 5 children diagnosed with ADHD	Set on Mars, the gameplay transforms each child into an astronaut on various adventures 3 male and 2 female	2 weekly sessions for a month
Kim et al [55]	Eye-contact game 40 children diagnosed with ADHD by DSM-5^d^ criteria	An eye contact game encourages children to recognize faces and improve attention and social interaction skills	30 min per session, 15 sessions total over 6 weeks
Martínez et al [56]	KAPEAN 15 children with ADHD	Children can interact with digital games using either a traditional mouse or hand gestures detected by a Leap Motion device	10 min for 4 months
Crepaldi et al [57]	Antonyms 16 boys aged 8–11, including 8 with ADHD and 8 controls	Players roleplay as superheroes in a realm where usual rules are inverted, needing to restrain impulsive actions to advance 16 male	45 min per session
Bul et al [58]	Plan-It Commander 170 children with ADHD diagnosis, aged 8-12 years, and their parents and teachers	The game engages players in a 10-mission online adventure, requiring specific skills to overcome challenges guided by a narrative 137 male and 33 female	65 min per session, 3 times per week, over 20 weeks
Castro and Huamanchahua [59]	Casa de Spots 35 children (19 with ADHD, 16 without ADHD)	The math learning game covers basic mathematics, including even and odd numbers, fractions, and geometric shapes	—
Celis et al [60]	Dilud 15 children with ADHD and their parents	Interactive and dynamic games designed to reinforce rote learning in children with ADHD employ the Tajima Cognitive Test method	10 min daily
Retalis et al [61]	Kinems 11 children diagnosed with ADHD	Touchless motion-based games use the player’s body movements and gestures for interaction 10 male and 1 female	30 min per session, 2-3 times per week for a month
Dovis et al [62]	Braingame Brian 89 children with a clinical diagnosis of ADHD, aged 8-12 years	Three minigames are designed to target distinct cognitive skills: working memory, inhibition, and cognitive flexibility 25 male and 6 female in full active; 22 male and 6 female in partially active; 24 male and 6 female in placebo	35-50 min per session, 5 times per week for 5 weeks
Gizatdinova et al [63]	PigScape 10 Children with ADHD	The game alternates between active and still phases to enhance impulse control, challenging children to mimic and hold postures	—
Ji et al [14]	Alchemist’s Treasure 30 children with ADHD (8-12 years).	Combines physical activity (running/jumping on a sensor-equipped board) with cognitive challenges (avoiding obstacles, collecting items) 26 male and 4 female	3 sessions per week, 50 min per day for 4 weeks
Ahmadi et al [64]	TARLAN 60 children (8-12 years): 40 with ADHD and 20 non-ADHD with social skills deficits	Simulation game with 40 scenarios using the SOCCSS model across four social contexts	8 sessions, 2 sessions per week, 20-30 minutes each, 3 phases
García-Redondo et al [65]	“Boogies Academy” and “Culbrain” 44 children with ADHD and SLD (6-16 years).	Each includes 10 subgames targeting specific intelligence (eg, logical-mathematical, spatial) 27 male and 17 female	28 sessions, 2 sessions per week, 10 min per session
Barba et al [66]	BRAVO 60 children (3-12 years old)	Three minigames, including spatial reasoning in virtual environments, obstacle avoidance with rule adherence and teamwork in a spaceship scenario	27 weeks
Kim et al [67]	NeuroWorld DTx^e^ 30 children (6-13 years old), 15 experimental, 15 control	Glass bridge recognition, spatial obstacle avoidance Remembering animal sounds and counts 23 male and 7 female	20 sessions, 30 minutes per day for 4 weeks

^a^ADHD: attention-deficit/hyperactivity disorder.

^b^Not available.

^c^VR: virtual reality.

^d^DSM-5: Diagnostic and Statistical Manual of Mental Disorders (Fifth Edition).

^e^DTx: digital therapeutics.

## Results

### Overview

**Figure 1:**
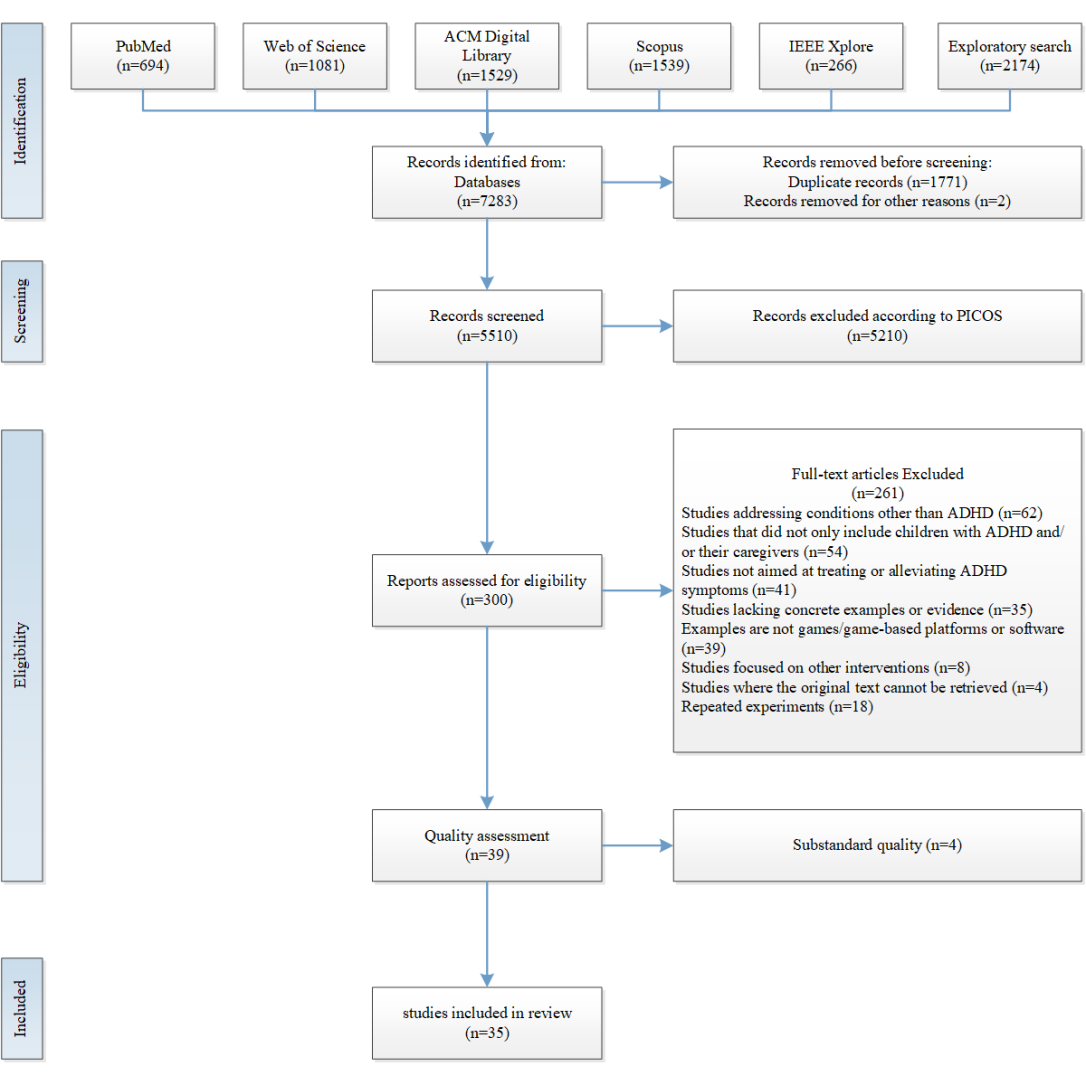
PRISMA (Preferred Reporting Items for Systematic Reviews and Meta-Analyses) flow diagram. ADHD: attention deficit hyperactivity disorder; PICOS: Participants, Intervention, Comparison, Outcomes, and Study.

The optimized search strategy identified 7283 additional records. After cross-validation with the exploration phase, with 1771 duplicates and 2 retracted articles excluded, yielding 5510 articles were aggregated using Zotero (version 6.0.36). After screening titles and abstracts, 300 articles met the inclusion criteria, and upon a complete reading of each article, 261 articles met the exclusion criteria. Finally, after a quality assessment excluded 4 articles, 35 publications were screened to fulfill the basic DTx criteria (Figure 1).

The 35 papers in our review were published in journals or conference proceedings across a variety of fields (based on the journal descriptions), as described in the journals. Of these, 14 were from interdisciplinary publications, 8 from computer science, 7 from psychology and medicine, 3 from education, and 3 from design. Among the game examples, wearables and sensors were used in 37% of papers (13/35), 37% (13/35) being 3D games and 63% (22/35) being 2D, and most were single player (31/35, 89%; Multimedia Appendix 4).

The selected papers encompassed 1408 participants, with sample sizes ranging from 1 to 206. Gender was recorded for 22 of the 35 trials, totaling 888 children, 660 male and 228 female. These data confirm the established sex disparity in ADHD prevalence, with male participants demonstrating significantly higher diagnosis rates than female participants [70]. Notable methodological limitations were identified, particularly regarding sample size constraints (median 20). Among the included studies, 28 provided intervention duration, and 64% (18) implemented interventions lasting fewer than 8 weeks, coupled with heterogeneous outcome measurement approaches. As some of the trials were quantitative research or qualitative research, there were issues of heterogeneity and bias or the results of competence enhancement were not rigorously quantified in the conclusions, we used a uniform use of descriptive analyses to elaborate on the findings (Table 4).

### Enjoyment

In total, 89% (31/35) of the studies investigated participants’ enjoyment of these serious games. The results on enjoyment were generally positive (Multimedia Appendix 5) [14,34-67], with 19% (6/31) of the trials including negative results on enjoyment. Children as the primary target group not only expressed their enjoyment through interviews and questionnaires, but some also directly expressed a desire to continue playing the game [35,54]. Although the games initially appeared very appealing, interest varied over time and could wane once children mastered them, leading to reduced enthusiasm [39,42]. However, children demonstrated consistently high satisfaction with the game intervention in most trials. In 45% (14/31) of the studies, player enjoyment was maintained by customizing the difficulty. Most measures of enjoyment were obtained through pre- and postintervention observations and assessments, satisfaction surveys, and electroencephalography (EEG) signal analyses. In particular, the game StiCap measures player enjoyment by testing the user experience with Smileyometer tests [46].

### Attention

Attention, identified as the most competency in need of improvement in children with ADHD, received focus in 80% (28/35) of the studies [14,34-56,64-67], with 2 studies emphasizing visual attention [41,65]. Among these games, 46% (13/28) of the studies focused on using carefully crafted game rules to promote attention [34-43,64,65,67]. Furthermore, 36% (10/28) of the studies concentrated on attentional training using various technologies [14,45-52,66]. Specifically, 7 studies used EEG headsets to capture brainwave data for observing attention levels [43,49-52,56,66], and 2 of these used attention levels, as measured by the brain-computer interface, to control gameplay [49,52]. 21% (6/28) of the studies focused on using in-game interaction design to motivate player focus, with 83% (5/6) using a Kinect sensor to recognize players’ movements or gestures and input this data into the virtual world to generate interactions [14,42,53,56,66], while 17% (1/6) applied design principles from the EMOINAD guide for mobile interfaces [54]. Notably, KAPEAN and BRAVO integrated wearable sensors, leveraging Kinect and electrocardiography to adapt therapy [56,66].

The studies consistently demonstrate significant improvements in attention spans among ADHD children across various gaming interventions (Multimedia Appendix 5) [14,34-67]. Changes in attention before and after the trials were assessed using various methods, the most common being standardized tests such as the Conners-3 scales, the Italian Battery for ADHD (BIA), the Attention Test for Elementary School Children (ATESC), and Swanson, Nolan, and Pelham Rating Scale, Version IV ratings, along with behavioral observations and electroencephalography signal analyses. Specifically, the game CIUDAD PUZZLE uses the perception of differences test (Faces-R) to measure improvements in attention performance [47].

### Hyperactivity-Impulsivity

In total, 29% (10/35) of studies focused on research related to hyperactivity-impulsivity; 30% (3/10) of studies focused on the interaction design and user experience [14,43,53], and 30% (3/10) of studies used virtual reality to create an immersive therapeutic environment for relief of hyperactivity-impulsivity [35,39,48]. Another 30% (3/10) of the studies focused on improving hyperactivity-impulsivity symptoms with counterintuitive game rules designed to stimulate impulsive responses, requiring players to inhibit these responses to succeed, and the game’s difficulty can be adjusted based on the child’s progress [57,63,67]. Hyperactivity-impulsivity symptom improvement was measured primarily by direct observation and decreased hyperactivity-impulsivity scores. Although some of the studies were not statistically emphasized, and 1 study reported no significant changes in hyperactivity-impulsivity symptoms (Evaluation of the Deficit of Attention and Hyperactivity scale) following a gaming intervention [65], it was possible to see improvements in inhibitory control in most studies.

### Social Skills

In total, 17% (6/35) of the studies focused on research related to social skills. In 3 of these studies, the single-player game Plan-It Commander, TARLAN, and BRAVO provide an in-game interactive environment where children learn and practice social skills through task challenges [58,64,66]. The eye-contact game requires participants to match and maintain eye contact with a virtual character [55]. The multiplayer game PigScape supports colocated gameplay, meaning that children play in pairs. This setup aims to create a learning environment in which children can support each other and enhance their social interactions and communication skills [63]. In particular, ADDventurous Rhythmical Planet offers the option to play alone or in collaboration with others, using a multiuser mode that fosters teamwork and communication. In addition to observational assessments, social skills can be scientifically assessed using the social skills rating system [34]. Although some of the trials were not statistically significant though not statistically significant, the results were generally positive.

### Motor Skills

In total, 7 (7/35, 20%) studies focused on motor skills. 57% (4/7) of these studies were designed to engage children in physical activities, using their body movements to interact with the game through a Kinect camera [42,53,61,63]. The remaining 3 studies used VR technology to train body coordination, specifically focusing on hand-eye and hand-foot coordination [39,43,66]. Children were observed to have enhanced hand-eye coordination because of interacting with the interface, but no significant changes in gross motor skills were noted, suggesting no targeted training or measurement for substantial motor skill development [39,42,53].

### Executive Functions

In total, 43% (15/35) of the studies focused on enhancing executive functioning, comprising 3 core functions: working memory, cognitive flexibility, and inhibitory control. Inhibitory control is discussed in the hyperactivity-impulsivity section. A total of 87% (13/15) of studies targeted working memory, primarily through repetitive practice in the games, with 2 studies focused on enhancing math skills [37,59]. Around 13% (2/15) of the studies aimed at enhancing children’s emotion regulation [38,56] were viewed as integrating cognitive flexibility and inhibitory control. Four of these studies enhanced children’s cognitive flexibility and problem-solving through virtual reality environments [35,39,48,66]. A total of 5 studies reported synthesizing improvements in executive function through multiple mini-games [39,48,58,64,66].

Most of the trials concluded that serious games had positive effects on executive functions, except for one report where enhanced cognitive flexibility and problem-solving abilities were observed in 1 participant through improved Wisconsin Card Sorting Test (WCST) scores, although the results for the other participants were not favorable [39]. Improvements in executive functioning are evaluated primarily through observational assessments by parents and teachers, and through scientific tests such as the Wechsler Memory Scale, Tower of London task (TOL), Behavioral Rating Inventory of Executive Function (BRIEF), and WCST. In addition, it is assessed by the scores achieved by the children during the game [60,62], such as behavioral tests like the Frankfurt Attention Inventory, Go/No-go [14], and through electroencephalography technology to monitor the children’s emotional and cognitive states during gameplay [56].

## Discussion

### Principal Findings

To our knowledge, this is not the inaugural systematic review in this field. However, it incorporates DTx guidelines to provide current and industry-standard insights, critically examining many original studies, serious games of various types, and outcomes. In addition, it addresses the multisymptom nature of ADHD, focusing on enhancements in social interactions, motor skills, and executive functions, extending beyond the core symptoms of inattention, and hyperactivity-impulsivity, to include children’s enjoyment of games. The review suggests that serious games hold great promise in the field of treatment for children with ADHD. However, findings were interpreted cautiously using descriptive analyses due to the wide variation in study design, sample size, targeting ability and duration, outcomes, and associated risks.

The results indicate that serious games may be beneficial for improving inattention, potentially serving as an effective alternative or supplement to traditional rehabilitation methods for key ADHD symptoms. Changes in EEG patterns, especially in the alpha and beta bands, support the effectiveness of serious games for enhancing attention [49]. Serious games provide a more engaging and digital format, enhancing enjoyment and promoting adherence among children [35,62]. This factor has been recognized as crucial for improving adherence to interventions [54]. The findings reveal that serious games use feedback mechanisms and engaging digital experience through game rules, technology, and interaction design to maximize participant motivation and minimize boredom [67,71,72]. Serious games can also be tailored to individual patient needs, resulting in more personalized and effective treatment. However, further research is necessary to examine the long-term effects of serious games on attention.

The integration of serious games into ADHD interventions provides a promising avenue for addressing hyperactivity-impulsivity symptoms in children. The aspect of impulsivity in ADHD, characterized by rash decision-making and difficulty in delaying gratification, can be addressed through games that require players to make calculated decisions to progress. Games that incorporate delay mechanisms or require the player to strategize can help in cultivating patience and strategic thinking, thus directly engaging with impulsivity control mechanisms. The games which focus on inhibitory control and cognitive flexibility, are reported to enhance the ability to suppress inappropriate motor responses and reduce impulsive behaviors in ADHD children [57,63]. Specifically, games that involve physical activity and require controlled responses have shown potential in managing these symptoms by improving motor skills and behavioral responses [39,48,53]. These findings are aligned with our observations where engagement in game-based tasks that demanded physical interaction not only helped in controlling impulsivity but also appeared to improve overall motor coordination [53] However, while digital games show promise, their effectiveness can vary based on the individual’s specific characteristics and the nature of the game mechanics. Games that require consistent engagement and provide immediate feedback, such as those with a narrative or competitive elements, may sustain attention better and lead to more significant improvements in hyperactive and impulsive behaviors [48,57].

The results of the study suggest that serious games may enhance the social skills of children with ADHD. Multiplayer gaming is not a requirement, games are primarily set in virtual environments that facilitate interaction or collaboration among peers, thereby aiding children with ADHD in developing their social skills. Most studies report consistent results, showing improvement in children’s social skills after using serious games, compared with the pre-experimental period. Children found the games engaging and expressed a desire to share their experiences with friends. However, when playing games with peers, children may experience frustration from not keeping up with friends for various reasons, leading to a loss of interest in continuing the game [34].

The current study does not find evidence to suggest a positive effect of serious games on the motor abilities of children with ADHD. However, the use of a Kinect sensor for full-body interaction during serious games is reported to be more engaging than traditional forms of exercise, enhancing fine motor skills, but there are no significant changes in gross motor skills [53]. In addition, limitations in motor skills enhancement results may stem from variations in measurement methods and study quality. Most studies concentrate on improving attention and inhibitory control in children with ADHD through exercise or physical activity, rather than sports instruction. Further research examining motor practice in serious games could shed more light on potential benefits for individuals with ADHD. This review also indicates that somatosensory digital systems, which use body movements and gestures as inputs, may achieve higher adherence rates than traditional games [63]. This underscores the importance of incorporating digital systems in serious gaming interventions, which could significantly affect their effectiveness. However, adherence rates may vary by population and specific intervention [73], necessitating further research to fully assess the impact of digital systems on adherence in serious gaming interventions.

Findings suggest that serious games may be beneficial and may improve executive functioning in children with ADHD. The studies in this review focused on working memory for executive functions, such as math skills, inhibitory control-related impulsivity symptoms, and emotion regulation. It should be emphasized that improvements in executive functioning may be influenced by a variety of factors and therefore may result in a lack of observed improvements [74]. Improvements in working memory can be more intuitively reflected in scores on games, and inhibitory control-related abilities are improved to some extent primarily by completing impulse-suppressing game challenges. Beyond this, it is not clear whether long-term interventions lead to significant improvements, which is consistent with the results of previous reviews [75].

The results of the current research on the enjoyment of serious games are generally positive, some of the studies that reported adverse outcomes involved difficult, reproducible, and device-related adverse events [44]. As the aim of these serious games is to improve various symptoms of ADHD, this often results in a relatively repetitive gameplay process. While games are initially appealing, participant interest varies over time, making compliance with the program one of the most significant challenges. [38,39,45]. Therefore, controlling the game’s difficulty is crucial, a game that is too difficult can frustrate children, leading them to give up [35,53,56]. However, games that are too easy may become uninteresting to children once mastered, leading to reduced enthusiasm unless new games are introduced. Of the 35 studies reviewed, 19 discussed customizing the game: 4 were customized for appearance only, while the remaining 15 involved adjusting levels and tasks according to the player’s performance. This suggests that game designers, to prevent boredom during gameplay, use progressively increasing levels of difficulty to stimulate children’s desire for a challenge [76]. For example, in “Alchemist’s Treasure,” exercise intensity was adjusted to 60%-80% of heart rate reserve based on individual metrics [14]. The difficulty levels in “Boogies Academy” and “Culbrain” were adapted based on age [65]. At the same time, the game uses gamification elements such as points, rewards, rankings, and levels as feedback mechanisms to minimize tedium [59,60]. Higher interactivity enhances player acceptance, and appropriate difficulty control can gain more favor among players. For example, in the game “The Secret Trail of the Moon,” among the 5 minigames, Teka Teki is highly interactive but also the most difficult, with a strict difficulty curve that impedes progress and may lead to a decline in initial motivation. In contrast, Kuburi, another reasonably difficult and interactive mini-game, received the highest usability score [35]. While serious games aim to address these issues through interesting experiences, they may still struggle to fully engage children who have severe attention deficits and impulsiveness, traits that are common in ADHD [49,50,56]. It appears that a suitable solution for this issue has not yet been identified and requires further examination by future research.

Recent advancements in serious games for ADHD incorporate diverse interventions. For example, cognitive training games strengthen attention and executive functions through adaptive memory and game tasks [35,48], whereas neurofeedback integrates real-time electrocardiography data to promote self-regulation [45,49,66]. Exergaming combines physical activity with gaming [14,53], while social simulation games use role-playing scenarios to enhance emotional awareness [58,64]. By blending cognitive, physical, and emotional strategies, serious games provide scalable, complementary tools that may amplify traditional ADHD interventions.

Although the probability of having more than 1 comorbid condition in people with ADHD is high [3], no in-depth or categorical studies of comorbid conditions were found in the included studies. Instead, researchers chose to prioritize a more representative sample of children with ADHD over a “pure” ADHD sample, which would be less generalizable [44]. Meanwhile, only one study categorized and discussed the subtypes of study participants to target treatment more effectively [35]. This oversight of comorbidities and subtypes may increase the difficulty of integrating the game into daily patient use.

Most studies in this review did not report safety outcomes associated with serious games. Of the 5 studies using VR technology, only 1 reported adverse effects, such as dizziness and virtual reality motion sickness [35]. Some children found the EEG headsets uncomfortable and encountered challenges in setting up and using this equipment effectively during gameplay [43]. Some children exhibited decreased performance in certain cognitive tasks after participating in game-based interventions, indicating that these games may not enhance all cognitive functions equally [39]. The common device-related adverse events were decreased frustration tolerance, headache, and irritability [44]. This indicates that potential adverse effects have not been fully identified, necessitating future studies to assess the safety outcomes of serious games and identify any possible adverse effects.

### Limitations

This review covers only original research in English from PubMed, Web of Science, Scopus, IEEE Xplore, and ACM Digital Library databases, published from January 2010 to January 2024. Although these publications furnish a robust foundation for academic research, they do not encompass the entirety of available literature, particularly that from disciplines outside our primary focus. Crucial studies published in other languages or indexed in databases predominant in other disciplines or geographical regions might not have been included. This limitation potentially omits crucial interdisciplinary insights and international perspectives that could influence the global understanding and treatment of ADHD. Future reviews could derive benefit from adopting a more inclusive approach, extending beyond English language publications to include a broader array of databases. Such inclusivity would enhance the global relevance and applicability of systematic reviews, ensuring a comprehensive synthesis that incorporates diverse methodological approaches and cultural perspectives. To the best of our ability, this limitation has been minimized by combining search strings and including more broadly defined terms in the keywords. Although the review incorporated DTx guidelines into the inclusion and exclusion criteria to comply with industry standards, the temporary deprioritization of certain fundamental DTx principles that do not currently apply to the research has also contributed to the limitations of the study.

In the review of 35 articles, bias was analyzed using 3 distinct methods, depending on the trial type: RCTs were assessed using the Cochrane Risk of Bias Tool 2.0 [77], nonrandomized quantitative studies were evaluated using the ROBINS-I [78], and qualitative studies were appraised using CASP checklists (Multimedia Appendix 6) [14,34-67,79]. RCTs typically showed low bias but were affected by deviations from intended interventions and subjective outcome assessments, raising concerns about generalizability. Quantitative studies faced biases from confounding factors and selective participant pools, lacking robust randomization and control. Qualitative research was compromised by nonrepresentative samples and insufficient methodological rigor, with potential skew from researcher and observer biases. These findings underscore the necessity for more rigorously designed studies with robust randomization, clear reporting, and diverse participant pools to ensure broader generalizability and more dependable conclusions. Despite limiting the study population to children with ADHD, the included studies were highly heterogeneous in terms of experiment type, game type, duration, sample size, targeting ability, assessment methods, and findings. Bias and heterogeneity necessitated descriptive rather than meta-analyses in this review. However, these studies initially demonstrate the potential for using serious games in ADHD treatment, providing data and insights for future research. They are expected to serve as a basis for obtaining clinical trial data and conducting more studies.

Although these limitations remain unresolved for the time being, the review still provides important implications for further research to enhance the evidence supporting serious games as digital therapeutics for enhancing ADHD treatment effectiveness in children.

### Conclusion

This review synthesized evidence from 35 studies on serious games as DTx in children with ADHD. The results indicated that the potential of serious games as a digital therapeutic modality for children with ADHD offers benefits in attention, hyperactivity-impulsivity symptoms, social skills, and executive functions. And interventions incorporating somatosensory input enhanced hand-eye coordination. While these findings align with growing interest in nonpharmacological alternative therapies, limitations like small sample sizes, short intervention durations, risk of bias, and heterogeneity of outcome measures temper their generalizability. Due to the highly heterogeneous nature of the included studies, the review opted for descriptive analysis over meta-analysis; yet, it still offers significant reference value for future research. To advance the field, future research should combine with DTx guidelines, and prioritize large-scale trials with standardized ADHD assessment protocols, rigorous experimental designs, and long-term efficacy evaluations. By bridging these gaps, serious games could become scalable, personalized interventions within children’s ADHD care frameworks.

## Data Availability

The datasets generated or analyzed during this study are available from the corresponding author on reasonable request.

## Appendix

Multimedia Appendix 1 PRISMA checklist.

Multimedia Appendix 2 Search strategies.

Multimedia Appendix 3 Descriptive analysis.

Multimedia Appendix 4 Quality assessment.

Multimedia Appendix 5 Results.

Multimedia Appendix 6 The risk of bias.

## Abbreviations

ADHD: attention-deficit/hyperactivity disorder
ATESC: Attention Test for Elementary School Children
BIA: Italian Battery for ADHD
BRIEF: Behavioral Rating Inventory of Executive Function
CASP: Critical Appraisal Skills Programme
DHI: digital health intervention
DTx: digital therapeutics
EEG: electroencephalography
ISO: International Organization for Standardization
MeSH: Medical Subject Headings
MVP: minimum viable product
PICOS: Participants, Intervention, Comparison, Outcomes, Study design
PRISMA: Preferred Reporting Items for Systematic Reviews and Meta-Analysis
RCT: randomized controlled trial
ROBINS-I: Cochrane Risk of Bias Tool for Non-Randomized Studies of Interventions
TOL: Tower of London task
WCST: Wisconsin Card Sorting Test
