# Supplementary material for: Effectiveness of Serious Games as Digital Therapeutics for Enhancing the Abilities of Children With Attention-Deficit/Hyperactivity Disorder (ADHD): Systematic Literature Review
Source: JMIR Serious Games. 2025 May 6;13:e60937. doi: 10.2196/60937 (PMC12093074; doi:10.2196/60937)
Supplement: Multimedia Appendix 2 [file games_v13i1e60937_app2.docx]

**Multimedia Appendix 5. Search strategies**

**Textbox S1. Search strategies.**

**PubMed:**

| Number | Search Query | Results |
| --- | --- | --- |
| #1 | "Attention Deficit Disorder with Hyperactivity" [MeSH] | 21,396 |
| #2 | "Hyperkinesis"[MeSH] | 984 |
| #3 | ADHD [tiab] | 25,248 |
| #4 | ADDH [tiab] | 25 |
| #5 | "Attention Deficit Disorders with Hyperactivity" [tiab] | 5 |
| #6 | "Attention Deficit Hyperactivity Disorder*"[tiab] | 23,894 |
| #7 | "Hyperkinetic Syndrome*"[tiab] | 39 |
| #8 | "Attention Deficit Disorder*" [tiab] | 1,113 |
| #9 | "Minimal Brain Dysfunction"[tiab] | 19 |
| #10 | #1 OR #2 OR #3 OR #4 OR #5 OR #6 OR #7 OR #8 OR #9 | 34,064 |
| #11 | "Video Games" [MeSH] | 6,739 |
| #12 | "Gamification" [MeSH] | 131 |
| #13 | "Games, Recreational"[MeSH] | 461 |
| #14 | game*[tiab] OR Gamif*[tiab] OR "Gaming"[tiab] OR serious games[tiab] OR serious game[tiab] OR game design[tiab] OR game based[tiab] OR game-based[tiab] | 58,609 |
| #15 | Educational game[tiab] OR learning game[tiab] OR "Exergaming" [MeSH] | 380 |
| #16 | "Cognitive training"[MeSH] OR "Cognitive Training"[tiab] | 4,174 |
| #17 | "Telemedicine" [MeSH] OR "mHealth"[tiab] | 43,069 |
| #18 | "Digital Health" [MeSH] | 153 |
| #19 | Digital health intervention[tiab] OR "DHI" [tiab] OR Digital Therapeutics[tiab] OR "DTx" [tiab] | 3,941 |
| #20 | #11 OR #12 OR #13 OR #14 OR #15 OR #16 OR #17 OR #18 OR #19 | 109,734 |
| #21 | #10 AND #20 | 694 |

Years of MeSH Terms::

Gamification[Mesh] (2022)

Exergaming[Mesh] (2022)

Cognitive Training[Mesh] (2023)

Digital Health[Mesh] (2024)

**WOS**

#1 TS=("Attention Deficit Disorder with Hyperactivity" OR Hyperkinesis OR ADHD OR ADDH OR "Attention Deficit Disorders with Hyperactivity" OR "Attention Deficit Hyperactivity Disorder*" OR "Hyperkinetic Syndrome*" OR "Attention Deficit Disorder*" OR "Minimal Brain Dysfunction")

42,970

#2 TS=("Video Games" OR Gamification OR "Recreational Games" OR game* OR Gamif* OR Gaming OR "serious game*" OR "game design" OR "game based" OR "game-based" OR "Educational game" OR "learning game" OR Exergaming OR "cognitive training" OR Telemedicine OR mHealth OR "Digital Health" OR "Digital health intervention" OR DHI OR "Digital Therapeutics" OR DTx)

307,867

#1 AND #2

Records retrieved:1081

**Scopus**

TITLE-ABS-KEY (("Attention Deficit Disorder with Hyperactivity" OR hyperkinesis OR adhd OR addh OR "Attention Deficit Disorders with Hyperactivity" OR "Attention Deficit Hyperactivity Disorder*" OR "Hyperkinetic Syndrome*" OR "Attention Deficit Disorder*" OR "Minimal Brain Dysfunction" ) ) AND TITLE-ABS-KEY ( ( "Video Games" OR gamification OR "Recreational Games" OR game* OR gamif* OR gaming OR "serious game*" OR "game design" OR "game based" OR "game-based" OR "Educational game" OR "learning game" OR exergaming OR "cognitive training" OR telemedicine OR mhealth OR "Digital Health" OR "Digital health intervention" OR dhi OR "Digital Therapeutics" OR dtx ) )

Records retrieved: 1539

**ACM**

[[Abstract: "attention deficit disorder with hyperactivity"] OR [Abstract: hyperkinesis] OR [Abstract: adhd] OR [Abstract: addh] OR [Abstract: "attention deficit disorders with hyperactivity"] OR [Abstract: "attention deficit hyperactivity disorder*"] OR [Abstract: "hyperkinetic syndrome*"] OR [Abstract: "attention deficit disorder*"] OR [Abstract: "minimal brain dysfunction"]] AND [[Abstract: "video games"] OR [Abstract: gamification] OR [Abstract: "recreational games"] OR [Abstract: game*] OR [Abstract: gamif*] OR [Abstract: gaming] OR [Abstract: "serious game*"] OR [Abstract: "game design"] OR [Abstract: "game based"] OR [Abstract: "game-based"] OR [Abstract: "educational game"] OR [Abstract: "learning game"] OR [Abstract: exergaming] OR [Abstract: "cognitive training"] OR [Abstract: telemedicine] OR [Abstract: mhealth] OR [Abstract: "digital health"] OR [Abstract: "digital health intervention"] OR [Abstract: dhi] OR [Abstract: "digital therapeutics"] OR [Abstract: dtx]]

Records retrieved: 1529

**IEEE**

("Attention Deficit Disorder with Hyperactivity" OR Hyperkinesis OR ADHD OR ADDH OR "Attention Deficit Disorders with Hyperactivity" OR "Attention Deficit Hyperactivity Disorder*" OR "Hyperkinetic Syndrome*" OR "Attention Deficit Disorder*" OR "Minimal Brain Dysfunction") AND ("Video Games" OR Gamification OR "Recreational Games" OR game* OR Gamif* OR Gaming OR "serious game*" OR "game design" OR "game based" OR "game-based" OR "Educational game" OR "learning game" OR Exergaming OR "cognitive training" OR Telemedicine OR mHealth OR "Digital Health" OR "Digital health intervention" OR DHI OR "Digital Therapeutics" OR DTx)

Records retrieved: 266
