# Supplementary material for: Effectiveness of Serious Games as Digital Therapeutics for Enhancing the Abilities of Children With Attention-Deficit/Hyperactivity Disorder (ADHD): Systematic Literature Review
Source: JMIR Serious Games. 2025 May 6;13:e60937. doi: 10.2196/60937 (PMC12093074; doi:10.2196/60937)
Supplement: Multimedia Appendix 4 [file games_v13i1e60937_app4.docx]

**Table S1.**

| Game reference | 1. Does the study's research design adhere to scientific and rational principles? | 2. Is the primary subject population of this study children with ADHD? | 3. Does the study discuss related works? | 4. Are there any significant flaws in the sample size or the quality of data used in this study? | 5. Does the study's outcome measurement focus on the improvement of core symptoms associated with ADHD, or does it evaluate aspects such as motor skills, executive functions, and social skills? | 6.Has the study provided a detailed description of the game-based interventions used? | 7.Do the interventions described in the study meet the fundamental definitions of Digital Therapeutics (DTX)? | Total score |
| --- | --- | --- | --- | --- | --- | --- | --- | --- |
| A 3D Rhythm-based Serious Game for Collaboration Improvement of Children with ADHD [1] | 1 | 1 | 1 | 0.5 | 1 | 1 | 1 | 0.93 |
| A Feasibility Study on the Effectiveness of a Full-Body Videogame Intervention for Decreasing Attention Deficit Hyperactivity Disorder Symptoms.[2] | 1 | 1 | 1 | 0.5 | 1 | 1 | 1 | 0.93 |
| A Virtual Reality Game (The Secret Trail of Moon) for Treating Attention-Deficit/Hyperactivity Disorder: Development and Usability Study [3] | 1 | 1 | 1 | 0 | 1 | 1 | 1 | 0.86 |
| Antonyms: A Computer Game to Improve Inhibitory Control of Impulsivity in Children with ADHD [4] | 1 | 1 | 1 | 0.5 | 1 | 1 | 1 | 0.93 |
| aTenDerAH: A Videogame to Support e-Learning Students with ADHD [5] | 0.5 | 0.5 | 1 | 0 | 0.5 | 1 | 0.5 | 0.57 |
| Behavioral Outcome Effects of Serious Gaming as an Adjunct to Treatment for Children With Attention-Deficit/Hyperactivity Disorder: A Randomized Controlled Trial [6] | 1 | 1 | 1 | 1 | 1 | 1 | 1 | 1.00 |
| Designing MIND PRO Working Memory Game and evaluating its effectiveness on working memory in ADHD children [7] | 1 | 1 | 1 | 0.5 | 1 | 1 | 1 | 0.93 |
| Developing an edutainment game, taboo!, for children with ADHD based on socially aware design and VCIA model [8] | 1 | 1 | 1 | 0.5 | 1 | 1 | 0.5 | 0.86 |
| Developing and feasibility testing of the Indonesian computer-based game prototype for children with attention deficit/hyperactivity disorder [9] | 1 | 1 | 1 | 0.5 | 1 | 1 | 0.5 | 0.86 |
| Development and Validation of a Gamified Videogame for Math Learning in Attention Deficit Hyperactivity Disorder Children (ADHD) [10] | 1 | 1 | 1 | 0.5 | 0.5 | 1 | 0.5 | 0.79 |
| Development of Serious Games for Neurorehabilitation of Children with ADHD through Neurofeedback [11] | 1 | 1 | 1 | 0 | 1 | 1 | 0.5 | 0.79 |
| Development of virtual reality rehabilitation games for children with attention-deficit hyperactivity disorder [12] | 1 | 1 | 1 | 0 | 1 | 1 | 0.5 | 0.79 |
| Dilud: A Mobile Application to Reinforce Rote Learning in Elementary School Children with Attention Deficit Hyperactivity Disorder [13] | 1 | 1 | 1 | 0.5 | 1 | 1 | 1 | 0.93 |
| DIVIDI2: Reinforcing Divided Attention in Children with AD/HD Through a Mobile Application [14] | 1 | 1 | 1 | 0 | 1 | 1 | 1 | 0.86 |
| Educational games based on distributed and tangible user interfaces to stimulate cognitive abilities in children with ADHD [15] | 1 | 1 | 1 | 0.5 | 1 | 1 | 1 | 0.93 |
| Effectiveness of a digital therapeutic as adjunct to treatment with medication in pediatric ADHD [16] | 1 | 1 | 1 | 1 | 1 | 1 | 1 | 1.00 |
| Empowering children with ADHD learning disabilities with the Kinems Kinect learning games [17] | 1 | 1 | 1 | 0.5 | 1 | 1 | 1 | 0.93 |
| Evaluating the ChillFish Biofeedback Game with Children with ADHD [18] | 0.5 | 1 | 1 | 0 | 0 | 1 | 0 | 0.50 |
| Exploring Learning in Near-Field Communication-Based Serious Games in Children Diagnosed with ADHD [19] | 1 | 1 | 1 | 0.5 | 1 | 1 | 0.5 | 0.86 |
| Eye-Contact Game Using Mixed Reality for the Treatment of Children With ADHD [20] | 1 | 1 | 1 | 1 | 1 | 1 | 1 | 1.00 |
| IAmHero: Preliminary Findings of an Experimental Study to Evaluate the Statistical Significance of an Intervention for ADHD Conducted through the Use of Serious Games in Virtual Reality. [21] | 1 | 1 | 1 | 0.5 | 1 | 1 | 1 | 0.93 |
| Improving Executive Functioning in Children with ADHD: Training Multiple Executive Functions within the Context of a Computer Game. A Randomized Double-Blind Placebo Controlled Trial [22] | 1 | 1 | 1 | 1 | 1 | 1 | 0.5 | 0.93 |
| Influence of a BCI neurofeedback videogame in children with ADHD: Quantifying the brain activity through an EEG signal processing dedicated toolbox [23] | 1 | 1 | 1 | 0 | 1 | 1 | 0.5 | 0.79 |
| KAPEAN: Understanding Affective States of Children with ADHD [24] | 1 | 1 | 1 | 0 | 0 | 1 | 0 | 0.57 |
| Keep Attention: A Personalized Serious Game for Attention Training [25] | 1 | 0.5 | 1 | 0 | 0 | 1 | 0 | 0.50 |
| Multisensory Virtual Game with Use of the Device Leap Motion to Improve the Lack of Attention in Children of 7–12 Years with ADHD [26] | 1 | 1 | 1 | 0.5 | 1 | 1 | 0.5 | 0.86 |
| NEUROBOT: A psycho-edutainment tool to perform neurofeedback training in children with ADHD [27] | 1 | 1 | 1 | 0 | 1 | 1 | 1 | 0.86 |
| Neurofeedback Based Attention Training for Children with ADHD [28] | 1 | 1 | 1 | 0 | 1 | 1 | 1 | 0.86 |
| Novel Interactive Eye-Tracking Game for Training Attention in Children With Attention-Deficit/Hyperactivity Disorder. [29] | 1 | 1 | 1 | 0.5 | 1 | 1 | 1 | 0.93 |
| PigScape: An embodied video game for cognitive peer-Training of impulse and behavior control in children with ADHD [30] | 1 | 1 | 1 | 0 | 1 | 1 | 1 | 0.86 |
| Quantifying Brain Activity State: EEG analysis of Background Music in A Serious Game on Attention of Children [31] | 1 | 0.5 | 1 | 0 | 1 | 1 | 1 | 0.79 |
| Towards the improvement of ADHD children through augmented reality serious games: Preliminary results [32] | 1 | 1 | 1 | 0 | 1 | 1 | 1 | 0.86 |
| User Experience Evaluation of the REEFOCUS ADHD Management Gaming System [33] | 1 | 1 | 1 | 0.5 | 1 | 1 | 1 | 0.93 |
| Neurofeedback Based Attention Training for Children with ADHD [28] | 0.5 | 1 | 1 | 0 | 1 | 0.5 | 0 | 0.57 |
| The Effects of Exergaming on Attention in Children With Attention Deficit/Hyperactivity Disorder: Randomized Controlled Trial. [34] | 1 | 1 | 1 | 0.5 | 1 | 1 | 1 | 0.93 |
| TARLAN: A Simulation Game to Improve Social Problem-Solving Skills of ADHD Children [35] | 1 | 1 | 1 | 0.5 | 1 | 1 | 1 | 0.93 |
| Serious Games and Their Effect Improving Attention in Students with Learning Disabilities [36] | 1 | 0.5 | 1 | 0.5 | 1 | 1 | 0.5 | 0.79 |
| BRAVO: A Gaming Environment for the Treatment of ADHD [37] | 1 | 1 | 1 | 0.5 | 0.5 | 1 | 0.5 | 0.79 |
| Adjuvant Therapy for Attention in Children with ADHD Using Game-Type Digital Therapy [38] | 1 | 1 | 1 | 0.5 | 1 | 1 | 0.5 | 0.86 |

### **References**

1. Giannaraki M, Moumoutzis N, Papatzanis Y, Kourkoutas E, Mania K. A 3D rhythm-based serious game for collaboration improvement of children with attention deficit hyperactivity disorder (ADHD). 2021. Presented at: IEEE Global Engineering Education Conference (EDUCON); 2021 April 21-23; Vienna, Austria. p. 1217-1225
2. Weerdmeester J, Cima M, Granic I, Hashemian Y, Gotsis M. A feasibility study on the effectiveness of a full-body videogame intervention for decreasing attention deficit hyperactivity disorder symptoms. Games Health J 2016; 5(4):258-269
3. Rodrigo-Yanguas M, Martin-Moratinos M, Menendez-Garcia A, Gonzalez-Tardon C, Royuela A, Blasco-Fontecilla H. A virtual reality game (The Secret Trail of Moon) for treating attention-deficit/hyperactivity disorder: development and usability study. JMIR Serious Games 2021; 9(3):e26824
4. Crepaldi M, Colombo V, Mottura S, Baldassini D, Sacco M, Cancer A, Antonietti A. Antonyms: A computer game to improve inhibitory control of impulsivity in children with attention deficit/hyperactivity disorder (ADHD). Information 2020; 11(4):230
5. Laura Mancera; Silvia Baldiris; Ramón Fabregat; Sergio Gomez; Carolina Mejia aTenDerAH: A Videogame to Support e-Learning Students with ADHD. Published in: 2017 IEEE 17th International Conference on Advanced Learning Technologies (ICALT).Date of Conference: 03-07 July 2017. DOI: 10.1109/ICALT.2017.157
6. Bul KCM, Kato PM, Van der Oord S, Danckaerts M, Vreeke LJ, Willems A, van Oers HJJ, Van Den Heuvel R, Birnie D, Van Amelsvoort TAMJ, Franken IHA, Maras A. Behavioral outcome effects of serious gaming as an adjunct to treatment for children with attention-deficit/hyperactivity disorder: A randomized controlled trial. J Med Internet Res 2016; 18(2):e26
7. Aghdam KS, Alavi MH. Designing MIND PRO working memory game and evaluating its effectiveness on working memory in ADHD children. 2019. Presented at: International Serious Games Symposium (ISGS); 2019 December 26; Tehran, Iran. p. 124-128
8. Batista BG, Rodrigues AFD, Miranda DM, Ishitani L, Nobre CN. Developing an edutainment game, taboo!, for children with ADHD based on socially aware design and VCIA model. 2022. Presented at: IHC '22: Proceedings of the 21st Brazilian Symposium on Human Factors in Computing Systems; 2022 October 17 - 21; Diamantina Brazil. p. 1-11
9. Wiguna T, Ismail RI, Kaligis F, Minayati K, Murtani BJ, Wigantara NA, Pradana K, Bahana R, Dirgantoro BP, Nugroho E. Developing and feasibility testing of the Indonesian computer-based game prototype for children with attention deficit/hyperactivity disorder. Heliyon 2021; 7(7):e07571
10. Castro R, Huamanchahua D. Development and validation of a gamified videogame for math learning in attention deficit hyperactivity disorder children (ADHD). 2021. Presented at: CEUR Workshop Proceedings; 2021 November 16-18; Chiclayo, Peru. p. 17-25
11. Machado FSV, Casagrande WD, Frizera A, Rocha FEM. Development of serious games for neurorehabilitation of children with attention-deficit/hyperactivity disorder through neurofeedback. 2019. Presented at: 18th Brazilian Symposium on Computer Games and Digital Entertainment (SBGames); 2019 October; Rio de Janeiro, Brazil. p. 91-97
12. Ou Y, Wang Y, Chang H, Yen S, Zheng Y, Lee B. Development of virtual reality rehabilitation games for children with attention-deficit hyperactivity disorder. J Ambient Intell Human Comput 2020; 11(11):5713-5720
13. Celis G, Casas M, Mauricio D, Santisteban J. Dilud: A mobile application to reinforce rote learning in elementary school children with attention deficit hyperactivity disorder. Int. J. Interact. Mob. Technol 2023; 17(06):62-80
14. Jácome V ID, Páez O JS, Cóllazos O CA, Fardoun HM. DIVIDI2: reinforcing divided attention in children with AD/HD through a mobile application. 2019. Presented at: REHAB '19: Proceedings of the 5th Workshop on ICTs for improving Patients Rehabilitation Research Techniques; 2019 September 11 - 13; Popayan Columbia. p. 106-110
15. de la Guía E, Lozano MD, Penichet VMR. Educational games based on distributed and tangible user interfaces to stimulate cognitive abilities in children with ADHD. Brit J Educational Tech 2014; 46(3):664-678
16. Kollins SH, Childress A, Heusser AC, Lutz J. Effectiveness of a digital therapeutic as adjunct to treatment with medication in pediatric ADHD. NPJ Digit Med 2021; 4(1):58
17. Retalis S, Korpa T, Skaloumpakas C, Boloudakis M, Kourakli M, Altanis G, Siameri F, Papadopoulou P, Lytra F, Pervanidou P. Empowering children with ADHD learning disabilities with the kinems kinect learning games. 2014. Presented at: 8th European Conference on Games Based Learning; 2014 October 9-10; Berlin, Germany. p. 28-39
18. Tobias Sonne, Mads Møller Jensen, Evaluating the ChillFish Biofeedback Game with Children with ADHD. IDC '16: Proceedings of the The 15th International Conference on Interaction Design and Children. Pages 529 – 534. https://doi.org/10.1145/2930674.2935981
19. Avila-Pesantez D, Santillán GS, Padilla N, Miriam AL, Arellano-Aucancela A. Exploring learning in near-field communication-based serious games in children diagnosed with ADHD. 2021. Presented at: Advances in Emerging Trends and Technologies; 2021 May 29-31; Quito, Ecuador. p. 314-324
20. Kim S, Ryu J, Choi Y, Kang Y, Li H, Kim K. Eye-contact game using mixed reality for the treatment of children with attention deficit hyperactivity disorder. IEEE Access 2020; 8:45996-46006
21. Schena A, Garotti R, D'Alise D, Giugliano S, Polizzi M, Trabucco V, Riccio MP, Bravaccio C. IAmHero: preliminary findings of an experimental study to evaluate the statistical significance of an intervention for ADHD conducted through the use of serious games in virtual reality. Int J Environ Res Public Health 2023; 20(4):0
22. Dovis S, Van der Oord S, Wiers RW, Prins PJM. Improving executive functioning in children with ADHD: training multiple executive functions within the context of a computer game. a randomized double-blind placebo controlled trial. PLoS One 2015; 10(4):e0121651
23. Blandon DZ, Munoz JE, Lopez DS, Gallo OH. Influence of a BCI neurofeedback videogame in children with ADHD. Quantifying the brain activity through an EEG signal processing dedicated toolbox. 2016. Presented at: IEEE 11th Colombian Computing Conference (CCC); 2016 September 27-30; Popayan, Colombia. p. 1-8
24. Fernando M, Claudia B, Nimrod G, Juan G. KAPEAN: understanding affective states of children with ADHD. Journal of Educational Technology & Society 2016; 19(2):18-28
25. Hocine, Nadia, Mohamed Ameur, Wafaa Ziani. "Keep Attention: A Personalized Serious Game for Attention Training." Proceedings of the 1st International Workshop on Intelligent Interfaces for Well-being at CHI 2019, CEUR-WS.org, 2019, pp. 1–8. Barcelona, SpainProceedings of the 3rd International Symposium on Gamification and Games for Learning (GamiLearn’19), Barcelona, Spain, 22-10-2019, published at http://ceurws.org
26. Capelo DC, Sánchez ME, Hurtado JS, Chicaiza DB. Multisensory virtual game with use of the device leap motion to improve the lack of attention in children of 7–12 years with ADHD. 2018. Presented at: Proceedings of the International Conference on Information Technology & Systems (ICITS 2018); 2018 January 10-12; Ecuador. p. 897-906
27. Vita S, Mennitto A. Neurobot: a psycho-edutainment tool to perform neurofeedback training in children with ADHD. 2019. Presented at: CEUR Workshop Proceedings; 2019 November 25-26; Naples; Italy.
28. Chen CL, Tang YW, Zhang NQ, Shin J. Neurofeedback based attention training for children with ADHD. 2017. Presented at: IEEE 8th International Conference on Awareness Science and Technology (iCAST); 2017 November 08-10; Taichung, Taiwan. p. 93-97
29. García-Baos A, D'Amelio T, Oliveira I, Collins P, Echevarria C, Zapata LP, Liddle E, Supèr H. Novel interactive eye-tracking game for training attention in children with attention-deficit/hyperactivity disorder. Prim Care Companion CNS Disord 2019; 21(4):0
30. Gizatdinova Y, Remizova V, Sand A, Sharma S, Rantanen K, Helminen T, Kylliäinen A. PigScape: An embodied video game for cognitive peer-training of impulse and behavior control in children with ADHD. 2022. Presented at: ASSETS '22: Proceedings of the 24th International ACM SIGACCESS Conference on Computers and Accessibility; 2022 October 23 - 26; Athens Greece. p. 1-4
31. Soysal ÖM, Kiran F, Chen J. Quantifying brain activity state: EEG analysis of background music in a serious game on attention of children. 2020. Presented at: 4th International Symposium on Multidisciplinary Studies and Innovative Technologies (ISMSIT); 2020 October 22-24; Istanbul, Turkey. p. 1-7
32. Avila-Pesantez D, Rivera LA, Vaca-Cardenas L, Aguayo S, Zuñiga L. Towards the improvement of ADHD children through augmented reality serious games: preliminary results. 2018. Presented at: IEEE Global Engineering Education Conference (EDUCON); 2018 April 17-20; Santa Cruz de Tenerife, Spain. p. 843-848
33. Kanellos T, Doulgerakis A, Georgiou E, Bessa M, Thomopoulos S, Vatakis A. User experience evaluation of the REEFOCUS ADHD management gaming system. 2019. Presented at: 4th International Conference on Smart and Sustainable Technologies (SpliTech); 2019 June 18-21; Split, Croatia. p. 1-6
34. Ji H, Wu S, Won J, Weng S, Lee S, Seo S, Park JJ. The effects of exergaming on attention in children with attention seficit/hyperactivity disorder: randomized controlled trial. JMIR Serious Games 2023; 11:e40438
35. Ahmadi A, Mitrovic A, Najmi B, Rucklidge J. TARLAN: a simulation game to improve social problem-solving skills of ADHD children. In: Artificial Intelligence in Education. Cham: Springer International Publishing; 2015. 328-337
36. García-Redondo P, García T, Areces D, Núñez JC, Rodríguez C. Serious games and their effect improving attention in students with learning disabilities. Int J Environ Res Public Health 2019; 16(14):2480
37. Barba MC, Covino A, De LV, DePaolis LT, D'Errico G, Di B. BRAVO: a gaming environment for the treatment of ADHD. In: Augmented Reality, Virtual Reality, and Computer Graphics. Cham: Springer International Publishing; 2019.
38. Kim S, Lee H, Lee H, Kim G, Song J. Adjuvant therapy for attention in children with ADHD using game-type digital therapy. Int J Environ Res Public Health 2022; 19(22):14982
