# Supplementary material for: Effectiveness of Serious Games as Digital Therapeutics for Enhancing the Abilities of Children With Attention-Deficit/Hyperactivity Disorder (ADHD): Systematic Literature Review
Source: JMIR Serious Games. 2025 May 6;13:e60937. doi: 10.2196/60937 (PMC12093074; doi:10.2196/60937)
Supplement: Multimedia Appendix 5 [file games_v13i1e60937_app5.docx]

| **Table S1.** Enjoyment. | | |
| --- | --- | --- |
| Reference | Results | Negative Results |
| A 3D Rhythm-based Serious Game for Collaboration Improvement of Children with ADHD [1] | Children enjoyed environment and multi-user mode, with no dropouts. |  |
| A Virtual Reality Game (The Secret Trail of Moon) for Treating Attention-Deficit/Hyperactivity Disorder: Development and Usability Study [2] | 86% of participants found the game satisfying, 83% wished to continue playing, appreciating the VR environment and story. | Challenges in higher difficulty levels of the "Teka Teki" minigame suggest areas for improvement. |
| Designing MIND PRO Working Memory Game and evaluating its effectiveness on working memory in ADHD children [3] | The children enjoyed the intervention sessions, no dropout or lack of engagement was reported . |  |
| Developing an edutainment game, taboo!, for children with ADHD based on socially aware design and VCIA model [4] | High engagement from game features such as customization, diverse environments, and a rewarding system that made mathematics interactive. | Feedback calls for enhancements in audio and gameplay challenges. |
| Developing and feasibility testing of the Indonesian computer-based game prototype for children with attention deficit/hyperactivity disorder [5] | Children reported the game as easy to play and enjoyable. | Task repetition was noted to become tedious over time. |
| Development of virtual reality rehabilitation games for children with attention-deficit hyperactivity disorder [6] | Participants initially found the VR games highly engaging and enjoyed the variety of tasks. | Allowing participants to select their preferred games in the final weeks restored interest, emphasizing the need for varied content to maintain motivation. |
| Multisensory Virtual Game with Use of the Device Leap Motion to Improve the Lack of Attention in Children of 7–12 Years with ADHD [7] | Leap Motion controller's multisensory interaction motivated children, increasing enthusiasm for learning. |  |
| Novel Interactive Eye-Tracking Game for Training Attention in Children With Attention-Deficit/Hyperactivity Disorder [8] | Eye-tracking interaction found engaging and enjoyable by children. |  |
| Towards the improvement of ADHD children through augmented reality serious games: Preliminary results [9] | Kinect's natural user interface increased children’s interest and participation in interactive problem-solving. |  |
| User Experience Evaluation of the REEFOCUS ADHD Management Gaming System [10] | MMR mode’s immersive elements were particularly enjoyable, enhancing engagement and interest. | The EEG headset was uncomfortable for younger children, impacting overall enjoyment. |
| Effectiveness of a digital therapeutic as adjunct to treatment with medication in pediatric ADHD [11] | Significant improvement in ADHD-related impairment and symptomatology in children aged 8-14, both on and off stimulant medication. | 18% of participants experienced device-related adverse events (AEs) during the trial, including decreased frustration tolerance, headaches, and irritability, all of mild or moderate severity. |
| Development of Serious Games for Neurorehabilitation of Children with ADHD through Neurofeedback [12] | It was noticed that patients who were more motivatedor who were able to follow the instructions were able tohave better results. |  |
| Educational games based on distributed and tangible user interfaces to stimulate cognitive abilities in children with ADHD [13] | Tangible interfaces in the system added a fun element, sustaining motivation and interest. |  |
| Exploring Learning in Near-Field Communication-Based Serious Games in Children Diagnosed with ADHD [14] | High levels of engagement and interest were reported. |  |
| IAmHero: Preliminary Findings of an Experimental Study to Evaluate the Statistical Significance of an Intervention for ADHD Conducted through the Use of Serious Games in Virtual Reality.[15] | The study suggests high engagement and positive feedback from the participants on the virtual reality intervention |  |
| Influence of a BCI neurofeedback videogame in children with ADHD: Quantifying the brain activity through an EEG signal processing dedicated toolbox [16] | Interactive and feedback-driven gameplay made sessions enjoyable and engaging. |  |
| NEUROBOT: A psycho-edutainment tool to perform neurofeedback training in children with ADHD [17] | High engagement and enjoyment were noted. |  |
| Neurofeedback Based Attention Training for Children with ADHD [18] | Game elements in neurofeedback training aimed to enhance enjoyment and motivation, showing effectiveness. |  |
| A Feasibility Study on the Effectiveness of a Full Body Videogame Intervention for Decreasing Attention Deficit Hyperactivity Disorder Symptoms [19] | The intervention game was evaluated as more difficult but was liked as much as the control game. |  |
| DIVIDI2: Reinforcing Divided Attention in Children with AD/HD Through a Mobile Application [20] | The game was well-received by participants, with high levels of engagement and positive feedba |  |
| Eye-Contact Game Using Mixed Reality for the Treatment of Children With ADHD [21] | High levels of enjoyment and engagement with the game were reported. |  |
| KAPEAN: Understanding Affective States of Children with ADHD [22] | The children showed high levels of engagement and positive responses to the interactive elements of KAPEAN. |  |
| Antonyms: A Computer Game to Improve Inhibitory Control of Impulsivity in Children with ADHD [23] | High engagement levels noted, with children enjoying the game and motivated to improve scores. |  |
| Behavioral Outcome Effects of Serious Gaming as an Adjunct to Treatment for Children With Attention-Deficit/Hyperactivity Disorder: A Randomized Controlled Trial [24] | High satisfaction with the serious game was reported by both parents and children |  |
| Development and Validation of a Gamified Videogame for Math Learning in Attention Deficit Hyperactivity Disorder Children (ADHD) [25] | Positive reception of gamified elements and interactive math challenges confirmed by a satisfaction survey. |  |
| Dilud: A Mobile Application to Reinforce Rote Learning in Elementary School Children with Attention Deficit Hyperactivity Disorder [26] | Children showed high levels of enjoyment and engagement with the Dilud app, as indicated by high usability and satisfaction ratings in the survey. |  |
| Empowering children with ADHD learning disabilities with the Kinems Kinect learning games [27] | Children demonstrated willingness to participate repeatedly, providing positive feedback post-sessions. |  |
| PigScape: An embodied video game for cognitive peer-Training of impulse and behavior control in children with ADHD [28] | No children dropped out during six gaming sessions. 70% felt time passed quickly and would recommend the training, most (80%) finding the game easy and enjoyable. |  |
| The Effects of Exergaming on Attention in Children With Attention Deficit/Hyperactivity Disorder: Randomized Controlled Trial. [29] | Higher retention in EXG and better engagement. |  |
| BRAVO: A Gaming Environment for the Treatment of ADHD [30] | Preliminary results note high patient involvement compared to traditional therapy. |  |
| Adjuvant Therapy for Attention in Children with ADHD Using Game-Type Digital Therapy [31] | 60% of children and 73% of parents reported satisfaction; 80% expressed willingness to re-participate. |  |
| **Table S2.** Attention. |  |  |
| Reference | Results | Negative Results |
| A 3D Rhythm-based Serious Game for Collaboration Improvement of Children with ADHD [1] | The game significantly enhanced attentional regulation in ADHD children via rhythm tasks. |  |
| A Virtual Reality Game (The Secret Trail of Moon) for Treating Attention-Deficit/Hyperactivity Disorder: Development and Usability Study [2] | "Smasher" minigame in TSTM boosted sustained attention, based on the Brown model of ADHD. |  |
| Designing MIND PRO Working Memory Game and evaluating its effectiveness on working memory in ADHD children [3] | The experiment showed selective and sustained attention improvements, confirmed by Wechsler Working Memory test data. |  |
| Developing an edutainment game, taboo!, for children with ADHD based on socially aware design and VCIA model [4] | During gameplay, improvements in focus and sustained attention were noted as children completed mathematical tasks. |  |
| Developing and feasibility testing of the Indonesian computer-based game prototype for children with attention deficit/hyperactivity disorder [5] | Engaging in focused tasks led to better sustained attention, evidenced by reduced CATPRS scores (p = 0.047). |  |
| Development of virtual reality rehabilitation games for children with attention-deficit hyperactivity disorder [6] | ATESC results showed significant enhancements in focused, sustained, and alternating attention, with all participants improving post-intervention. |  |
| Multisensory Virtual Game with Use of the Device Leap Motion to Improve the Lack of Attention in Children of 7–12 Years with ADHD [7] | Improved sustained attention observed in ADHD participants, shown by better performance in organizing and placing objects over time. |  |
| Novel Interactive Eye-Tracking Game for Training Attention in Children With Attention-Deficit/Hyperactivity Disorder. [8] | Enhanced attentional control in ADHD children was noted, with improved performance in sustained and selective tasks. |  |
| Towards the improvement of ADHD children through augmented reality serious games: Preliminary results [9] | Faster completion times and higher accuracy in ATHYNOS game tasks indicated improved selective and sustained attention. |  |
| User Experience Evaluation of the REEFOCUS ADHD Management Gaming System [10] | Cognitive skill mini-games improved sustained and selective attention in delay aversion and inhibitory control tasks. |  |
| Effectiveness of a digital therapeutic as adjunct to treatment with medication in pediatric ADHD [11] | Improved IRS and ADHD-RS scores indicated better management of attention deficits in ADHD-related impairments. |  |
| Development of Serious Games for Neurorehabilitation of Children with ADHD through Neurofeedback [12] | Neurofeedback-driven game tasks enhanced sustained attention by increasing attentional control. |  |
| Educational games based on distributed and tangible user interfaces to stimulate cognitive abilities in children with ADHD [13] | Interaction with tangible user interfaces in the game effectively boosted children's focus on tasks. |  |
| Exploring Learning in Near-Field Communication-Based Serious Games in Children Diagnosed with ADHD [14] | An average 2.9 increase in learning performance scores indicated improved attention in children. |  |
| IAmHero: Preliminary Findings of an Experimental Study to Evaluate the Statistical Significance of an Intervention for ADHD Conducted through the Use of Serious Games in Virtual Reality. [15] | Decreased inattention scores on Conners-3 scales suggested attention improvements. |  |
| Influence of a BCI neurofeedback videogame in children with ADHD: Quantifying the brain activity through an EEG signal processing dedicated toolbox [16] | Increased attention levels and better task completion metrics during gameplay indicated improved attention regulation. |  |
| NEUROBOT: A psycho-edutainment tool to perform neurofeedback training in children with ADHD [17] | Enhanced focus during sessions evidenced by EEG data and robot control performance metrics. |  |
| Neurofeedback Based Attention Training for Children with ADHD [18] | Significant attention improvements, including enhanced vigilance, sustained, divided, and selective attention. |  |
| Quantifying Brain Activity State: EEG analysis of Background Music in A Serious Game on Attention of Children [19] | Increased Beta-level brain activity linked to higher attention and alertness during Mozart music exposure. |  |
| A Feasibility Study on the Effectiveness of a Full-Body Videogame Intervention for Decreasing Attention Deficit Hyperactivity Disorder Symptoms [20] | Both groups improved in attention, but the intervention group showed a greater decrease in ADHD symptoms. |  |
| DIVIDI2: Reinforcing Divided Attention in Children with AD/HD Through a Mobile Application [21] | The game effectively reinforced divided attention by requiring simultaneous focus on multiple stimuli. |  |
| Eye-Contact Game Using Mixed Reality for the Treatment of Children With ADHD [22] | Lower error rates in attention tests indicated improved focus and task completion. |  |
| KAPEAN: Understanding Affective States of Children with ADHD [23] | KAPEAN project activities designed to enhance cognitive engagement positively affected attention in ADHD children. |  |
| The Effects of Exergaming on Attention in Children With Attention Deficit/Hyperactivity Disorder: Randomized Controlled Trial. [31] | Both exergaming and bicycle exercise significantly improved selective and continuous attention, with exergaming showing greater improvements in N2 amplitude. |  |
| TARLAN: A Simulation Game to Improve Social Problem-Solving Skills of ADHD Children [32] | ADHD children's focus matched non-ADHD children by session 3 |  |
| Serious Games and Their Effect Improving Attention in Students with Learning Disabilities [33] | Significant improvement in visual attention (D2 test) in the experimental group. |  |
| BRAVO: A Gaming Environment for the Treatment of ADHD [34] | Games improved sustained attention and selective attention. |  |
| Adjuvant Therapy for Attention in Children with ADHD Using Game-Type Digital Therapy [35] | Significant improvement in auditory selective attention (sensitivity coefficient increased, p = 0.037). Flanker task response style improved in the experimental group (p = 0.020). K-ARS attention deficit scores decreased significantly (p = 0.018). |  |
| **Table S3.** Hyperactivity-impulsivity. |  |  |
| Reference | Results | Negative Results |
| A Virtual Reality Game (The Secret Trail of Moon) for Treating Attention-Deficit/Hyperactivity Disorder: Development and Usability Study [2] | "Smasher" minigame enhances inhibitory control, helping manage impulsive behaviors through structured tasks, though not statistically emphasized. |  |
| Development of virtual reality rehabilitation games for children with attention-deficit hyperactivity disorder [6] | All three participants showed lower hyperactivity/impulsivity scores on the SNAP-IV scale, indicating symptom reduction. |  |
| User Experience Evaluation of the REEFOCUS ADHD Management Gaming System [10] | Increased focus and decreased impulsive responses during gameplay indicated improvements in inhibitory control. |  |
| IAmHero: Preliminary Findings of an Experimental Study to Evaluate the Statistical Significance of an Intervention for ADHD Conducted through the Use of Serious Games in Virtual Reality. [15] | Decreased hyperactivity/impulsivity scores demonstrated improved management of impulsivity and hyperactive behavior. |  |
| A Feasibility Study on the Effectiveness of a Full-Body Videogame Intervention for Decreasing Attention Deficit Hyperactivity Disorder Symptoms. [20] | Marginally significant interaction indicates impulsivity improvements in the intervention group, with reduced hyperactivity-impulsivity symptoms compared to control. |  |
| Antonyms: A Computer Game to Improve Inhibitory Control of Impulsivity in Children with ADHD [24] | Improved response control in children was observed as they played the game, reflecting better inhibitory control. |  |
| PigScape: An embodied video game for cognitive peer-Training of impulse and behavior control in children with ADHD [30] | Enhanced abilities were measured by heart rate variability. |  |
| The Effects of Exergaming on Attention in Children With Attention Deficit/Hyperactivity Disorder: Randomized Controlled Trial. [31] | Greater N2 amplitude improvements in EXG suggest enhanced inhibitory control. |  |
| Serious Games and Their Effect Improving Attention in Students with Learning Disabilities [33] |  | No significant changes in hyperactivity/impulsivity symptoms (EDAH scale). |
| Adjuvant Therapy for Attention in Children with ADHD Using Game-Type Digital Therapy [35] | K-ARS hyperactivity scores decreased (p = 0.004). CGI-Severity scores improved in the experimental group (p = 0.028). |  |
| **Table S4.** Social skills. |  |  |
| Reference | Results | Negative Results |
| A 3D Rhythm-based Serious Game for Collaboration Improvement of Children with ADHD [1] | Enhanced social skills and collaboration noted as participants synchronized and cooperated in multiplayer rhythmical tasks. |  |
| Eye-Contact Game Using Mixed Reality for the Treatment of Children With ADHD [22] | The game targeted eye-contact, vital for social interactions, though no direct measurement of overall social skills improvement was reported. |  |
| Behavioral Outcome Effects of Serious Gaming as an Adjunct to Treatment for Children With Attention-Deficit/Hyperactivity Disorder: A Randomized Controlled Trial [25] | Improvements in social skills were noted within groups, but between-group effects were not significant. |  |
| PigScape: An embodied video game for cognitive peer-Training of impulse and behavior control in children with ADHD [30] | Co-located gameplay facilitated pair interactions, designed to enhance peer communication, though not directly explicitly quantified. |  |
| TARLAN: A Simulation Game to Improve Social Problem-Solving Skills of ADHD Children [32] | Significant improvement for ADHD children (t=3.16, p<0.01); ADHD-Com group outperformed ADHD-Psy group; targets five key social skills. |  |
| BRAVO: A Gaming Environment for the Treatment of ADHD [34] | Space Travel Trainer involved team interactions and collaborative problem-solving. |  |
| **Table S5.** Motor skills |  |  |
| Reference | Results | Negative Results |
| Development of virtual reality rehabilitation games for children with attention-deficit hyperactivity disorder [6] | The games focused on improving hand-eye and hand-foot coordination, though motor skill enhancements were not explicitly quantified. |  |
| Towards the improvement of ADHD children through augmented reality serious games: Preliminary results [9] | Enhanced hand-eye coordination was observed in drag-and-drop tasks using the Kinect interface. |  |
| User Experience Evaluation of the REEFOCUS ADHD Management Gaming System [10] | Motor coordination improvements were significant in the Multisensory Mixed Reality (MMR) mode, featuring tangible user interfaces and immersive interactions. |  |
| A Feasibility Study on the Effectiveness of a Full-Body Videogame Intervention for Decreasing Attention Deficit Hyperactivity Disorder Symptoms. [20] | Both groups improved equally in fine motor skills, but no significant changes in gross motor skills were noted. |  |
| Empowering children with ADHD learning disabilities with the Kinems Kinect learning games [28] | Kinect-based games led to enhanced motor skills, requiring physical responses and improved coordination. |  |
| PigScape: An embodied video game for cognitive peer-Training of impulse and behavior control in children with ADHD [30] | Active gameplay, involving physical movements such as jumping and maintaining balance, was expected to improve motor skills and postural control, although specific results were not detailed. |  |
| BRAVO: A Gaming Environment for the Treatment of ADHD [34] | Kinect-based Infinite Runner and HTC Vive games require body movements, improving coordination. |  |
| **Table S6.** Executive functions. |  |  |
| Reference | Results | Negative Results |
| A Virtual Reality Game (The Secret Trail of Moon) for Treating Attention-Deficit/Hyperactivity Disorder: Development and Usability Study [2] | "Teka Teki" and "Enigma" minigame enhance planning, working memory, and cognitive flexibility, though not directly explicitly quantified. |  |
| Designing MIND PRO Working Memory Game and evaluating its effectiveness on working memory in ADHD children [3] | Significant improvement in working memory shown through pre-test, post-test, and follow-up evaluations. |  |
| Developing an edutainment game, taboo!, for children with ADHD based on socially aware design and VCIA model [4] | Children showed improvements in problem-solving, organization, and planning abilities. |  |
| Developing and feasibility testing of the Indonesian computer-based game prototype for children with attention deficit/hyperactivity disorder [5] | Improvements in executive function domains were significant, including working memory (BRIEF-Working Memory T-score reduction, p = 0.02), initiation (p = 0.008), and organization (BRIEF-Organization of Materials, p = 0.04). |  |
| Development of virtual reality rehabilitation games for children with attention-deficit hyperactivity disorder [6] |  | One participant showed enhanced cognitive flexibility and problem-solving through improved WCST scores, while others had less favorable outcomes. |
| IAmHero: Preliminary Findings of an Experimental Study to Evaluate the Statistical Significance of an Intervention for ADHD Conducted through the Use of Serious Games in Virtual Reality. [15] | Scores exhibited improvement in tests related to problem-solving and executive functions |  |
| KAPEAN: Understanding Affective States of Children with ADHD [23] | Enhanced executive functioning observed in task engagement and cognitive activities during game play, although not explicitly quantified in terms of traditional executive function tests. |  |
| Behavioral Outcome Effects of Serious Gaming as an Adjunct to Treatment for Children With Attention-Deficit/Hyperactivity Disorder: A Randomized Controlled Trial [25] | Observed improvements in planning/organizing skills, though not statistically significant between groups. |  |
| Development and Validation of a Gamified Videogame for Math Learning in Attention Deficit Hyperactivity Disorder Children (ADHD) [26] | The game supported executive function development through rule following, problem solving, and task management within gameplay. |  |
| Dilud: A Mobile Application to Reinforce Rote Learning in Elementary School Children with Attention Deficit Hyperactivity Disorder [27] | The Dilud app significantly improved children's working memory, with scores increasing from 84 to 103 on the WISC-V scale. |  |
| Empowering children with ADHD learning disabilities with the Kinems Kinect learning games [28] | Significant improvements in task completion and problem-solving abilities. |  |
| Improving Executive Functioning in Children with ADHD: Training Multiple Executive Functions within the Context of a Computer Game. A Randomized Double-Blind Placebo Controlled Trial [29] | Significant improvements in visuospatial STM(Short-Term Memory) and WM(Working Memory) in the full-active condition; inhibition improved in both full-active and partially-active conditions. |  |
| The Effects of Exergaming on Attention in Children With Attention Deficit/Hyperactivity Disorder: Randomized Controlled Trial. [31] | Improved self-control (FAIR Q index) and persistent attention (FAIR C index) in both groups. |  |
| TARLAN: A Simulation Game to Improve Social Problem-Solving Skills of ADHD Children [32] | 82.5% of ADHD children successfully solved self-identified problems; improved analysis of social situations. |  |
| BRAVO: A Gaming Environment for the Treatment of ADHD [34] | Space Travel Trainer emphasizes action planning and decision-making. |  |

### **References**

1. Giannaraki M, Moumoutzis N, Papatzanis Y, Kourkoutas E, Mania K. A 3D rhythm-based serious game for collaboration improvement of children with attention deficit hyperactivity disorder (ADHD). 2021. Presented at: IEEE Global Engineering Education Conference (EDUCON); 2021 April 21-23; Vienna, Austria. p. 1217-1225
2. Rodrigo-Yanguas M, Martin-Moratinos M, Menendez-Garcia A, Gonzalez-Tardon C, Royuela A, Blasco-Fontecilla H. A virtual reality game (The Secret Trail of Moon) for treating attention-deficit/hyperactivity disorder: development and usability study. JMIR Serious Games 2021; 9(3):e26824
3. Aghdam KS, Alavi MH. Designing MIND PRO working memory game and evaluating its effectiveness on working memory in ADHD children. 2019. Presented at: International Serious Games Symposium (ISGS); 2019 December 26; Tehran, Iran. p. 124-128
4. Batista BG, Rodrigues AFD, Miranda DM, Ishitani L, Nobre CN. Developing an edutainment game, taboo!, for children with ADHD based on socially aware design and VCIA model. 2022. Presented at: IHC '22: Proceedings of the 21st Brazilian Symposium on Human Factors in Computing Systems; 2022 October 17 - 21; Diamantina Brazil. p. 1-11
5. Wiguna T, Ismail RI, Kaligis F, Minayati K, Murtani BJ, Wigantara NA, Pradana K, Bahana R, Dirgantoro BP, Nugroho E. Developing and feasibility testing of the Indonesian computer-based game prototype for children with attention deficit/hyperactivity disorder. Heliyon 2021; 7(7):e07571
6. Ou Y, Wang Y, Chang H, Yen S, Zheng Y, Lee B. Development of virtual reality rehabilitation games for children with attention-deficit hyperactivity disorder. J Ambient Intell Human Comput 2020; 11(11):5713-5720
7. Capelo DC, Sánchez ME, Hurtado JS, Chicaiza DB. Multisensory virtual game with use of the device leap motion to improve the lack of attention in children of 7–12 years with ADHD. 2018. Presented at: Proceedings of the International Conference on Information Technology & Systems (ICITS 2018); 2018 January 10-12; Ecuador. p. 897-906
8. García-Baos A, D'Amelio T, Oliveira I, Collins P, Echevarria C, Zapata LP, Liddle E, Supèr H. Novel interactive eye-tracking game for training attention in children with attention-deficit/hyperactivity disorder. Prim Care Companion CNS Disord 2019; 21(4):0
9. Avila-Pesantez D, Rivera LA, Vaca-Cardenas L, Aguayo S, Zuñiga L. Towards the improvement of ADHD children through augmented reality serious games: preliminary results. 2018. Presented at: IEEE Global Engineering Education Conference (EDUCON); 2018 April 17-20; Santa Cruz de Tenerife, Spain. p. 843-848
10. Kanellos T, Doulgerakis A, Georgiou E, Bessa M, Thomopoulos S, Vatakis A. User experience evaluation of the REEFOCUS ADHD management gaming system. 2019. Presented at: 4th International Conference on Smart and Sustainable Technologies (SpliTech); 2019 June 18-21; Split, Croatia. p. 1-6
11. Kollins SH, Childress A, Heusser AC, Lutz J. Effectiveness of a digital therapeutic as adjunct to treatment with medication in pediatric ADHD. NPJ Digit Med 2021; 4(1):58
12. Machado FSV, Casagrande WD, Frizera A, Rocha FEM. Development of serious games for neurorehabilitation of children with attention-deficit/hyperactivity disorder through neurofeedback. 2019. Presented at: 18th Brazilian Symposium on Computer Games and Digital Entertainment (SBGames); 2019 October; Rio de Janeiro, Brazil. p. 91-97
13. de la Guía E, Lozano MD, Penichet VMR. Educational games based on distributed and tangible user interfaces to stimulate cognitive abilities in children with ADHD. Brit J Educational Tech 2014; 46(3):664-678
14. Avila-Pesantez D, Santillán GS, Padilla N, Miriam AL, Arellano-Aucancela A. Exploring learning in near-field communication-based serious games in children diagnosed with ADHD. 2021. Presented at: Advances in Emerging Trends and Technologies; 2021 May 29-31; Quito, Ecuador. p. 314-324
15. Schena A, Garotti R, D'Alise D, Giugliano S, Polizzi M, Trabucco V, Riccio MP, Bravaccio C. IAmHero: preliminary findings of an experimental study to evaluate the statistical significance of an intervention for ADHD conducted through the use of serious games in virtual reality. Int J Environ Res Public Health 2023; 20(4):0
16. Blandon DZ, Munoz JE, Lopez DS, Gallo OH. Influence of a BCI neurofeedback videogame in children with ADHD. Quantifying the brain activity through an EEG signal processing dedicated toolbox. 2016. Presented at: IEEE 11th Colombian Computing Conference (CCC); 2016 September 27-30; Popayan, Colombia. p. 1-8
17. Vita S, Mennitto A. Neurobot: a psycho-edutainment tool to perform neurofeedback training in children with ADHD. 2019. Presented at: CEUR Workshop Proceedings; 2019 November 25-26; Naples; Italy.
18. Chen CL, Tang YW, Zhang NQ, Shin J. Neurofeedback based attention training for children with ADHD. 2017. Presented at: IEEE 8th International Conference on Awareness Science and Technology (iCAST); 2017 November 08-10; Taichung, Taiwan. p. 93-97
19. Soysal ÖM, Kiran F, Chen J. Quantifying brain activity state: EEG analysis of background music in a serious game on attention of children. 2020. Presented at: 4th International Symposium on Multidisciplinary Studies and Innovative Technologies (ISMSIT); 2020 October 22-24; Istanbul, Turkey. p. 1-7
20. Weerdmeester J, Cima M, Granic I, Hashemian Y, Gotsis M. A feasibility study on the effectiveness of a full-body videogame intervention for decreasing attention deficit hyperactivity disorder symptoms. Games Health J 2016; 5(4):258-269
21. Jácome V ID, Páez O JS, Cóllazos O CA, Fardoun HM. DIVIDI2: reinforcing divided attention in children with AD/HD through a mobile application. 2019. Presented at: REHAB '19: Proceedings of the 5th Workshop on ICTs for improving Patients Rehabilitation Research Techniques; 2019 September 11 - 13; Popayan Columbia. p. 106-110
22. Kim S, Ryu J, Choi Y, Kang Y, Li H, Kim K. Eye-contact game using mixed reality for the treatment of children with attention deficit hyperactivity disorder. IEEE Access 2020; 8:45996-46006
23. Fernando M, Claudia B, Nimrod G, Juan G. KAPEAN: understanding affective states of children with ADHD. Journal of Educational Technology & Society 2016; 19(2):18-28
24. Crepaldi M, Colombo V, Mottura S, Baldassini D, Sacco M, Cancer A, Antonietti A. Antonyms: A computer game to improve inhibitory control of impulsivity in children with attention deficit/hyperactivity disorder (ADHD). Information 2020; 11(4):230
25. Bul KCM, Kato PM, Van der Oord S, Danckaerts M, Vreeke LJ, Willems A, van Oers HJJ, Van Den Heuvel R, Birnie D, Van Amelsvoort TAMJ, Franken IHA, Maras A. Behavioral outcome effects of serious gaming as an adjunct to treatment for children with attention-deficit/hyperactivity disorder: A randomized controlled trial. J Med Internet Res 2016; 18(2):e26
26. Castro R, Huamanchahua D. Development and validation of a gamified videogame for math learning in attention deficit hyperactivity disorder children (ADHD). 2021. Presented at: CEUR Workshop Proceedings; 2021 November 16-18; Chiclayo, Peru. p. 17-25
27. Celis G, Casas M, Mauricio D, Santisteban J. Dilud: A mobile application to reinforce rote learning in elementary school children with attention deficit hyperactivity disorder. Int. J. Interact. Mob. Technol 2023; 17(06):62-80
28. Retalis S, Korpa T, Skaloumpakas C, Boloudakis M, Kourakli M, Altanis G, Siameri F, Papadopoulou P, Lytra F, Pervanidou P. Empowering children with ADHD learning disabilities with the kinems kinect learning games. 2014. Presented at: 8th European Conference on Games Based Learning; 2014 October 9-10; Berlin, Germany. p. 28-39
29. Dovis S, Van der Oord S, Wiers RW, Prins PJM. Improving executive functioning in children with ADHD: training multiple executive functions within the context of a computer game. a randomized double-blind placebo controlled trial. PLoS One 2015; 10(4):e0121651
30. Gizatdinova Y, Remizova V, Sand A, Sharma S, Rantanen K, Helminen T, Kylliäinen A. PigScape: An embodied video game for cognitive peer-training of impulse and behavior control in children with ADHD. 2022. Presented at: ASSETS '22: Proceedings of the 24th International ACM SIGACCESS Conference on Computers and Accessibility; 2022 October 23 - 26; Athens Greece. p. 1-4
31. Ji H, Wu S, Won J, Weng S, Lee S, Seo S, Park JJ. The effects of exergaming on attention in children with attention deficit/hyperactivity disorder: randomized controlled trial. JMIR Serious Games 2023; 11:e40438
32. Ahmadi A, Mitrovic A, Najmi B, Rucklidge J. TARLAN: a simulation game to improve social problem-solving skills of ADHD children. In: Artificial Intelligence in Education. Cham: Springer International Publishing; 2015. 328-337
33. García-Redondo P, García T, Areces D, Núñez JC, Rodríguez C. Serious games and their effect improving attention in students with learning disabilities. Int J Environ Res Public Health 2019; 16(14):2480
34. Barba MC, Covino A, De LV, DePaolis LT, D'Errico G, Di B. BRAVO: a gaming environment for the treatment of ADHD. In: Augmented Reality, Virtual Reality, and Computer Graphics. Cham: Springer International Publishing; 2019.
35. Kim S, Lee H, Lee H, Kim G, Song J. Adjuvant therapy for attention in children with ADHD using game-type digital therapy. Int J Environ Res Public Health 2022; 19(22):14982
