# Supplementary material for: Effectiveness of Serious Games as Digital Therapeutics for Enhancing the Abilities of Children With Attention-Deficit/Hyperactivity Disorder (ADHD): Systematic Literature Review
Source: JMIR Serious Games. 2025 May 6;13:e60937. doi: 10.2196/60937 (PMC12093074; doi:10.2196/60937)
Supplement: Multimedia Appendix 6 [file games_v13i1e60937_app6.docx]

| **Table S1.** RCT. | | | | | | | |
| --- | --- | --- | --- | --- | --- | --- | --- |
| the Cochrane Risk of Bias Tool 2.0 (RoB 2.0) | Bias arising from the randomization process | Bias due to deviations from intendedinterventions | Bias due to missing outcome data | Bias in measurement of the outcome | Bias in selection of the reported result | Overall risk of bias |  |
| A Virtual Reality Game (The Secret Trail of Moon) for Treating Attention-Deficit/Hyperactivity Disorder: Development and Usability Study [1] | Low | Some concerns | Low | Some concerns | Low | Some concerns |  |
| User Experience Evaluation of the REEFOCUS ADHD Management Gaming System [2] | Some concerns | Low | Low | Some concerns | Some concerns | Some concerns |  |
| IAmHero: Preliminary Findings of an Experimental Study to Evaluate the Statistical Significance of an Intervention for ADHD Conducted through the Use of Serious Games in Virtual Reality. [3] | Low | Low | Some concerns | Low | Low | Low |  |
| A Feasibility Study on the Effectiveness of a Full-Body Videogame Intervention for Decreasing Attention Deficit Hyperactivity Disorder Symptoms. [4] | Low | Low | Low | Low | Some concerns | Low |  |
| Behavioral Outcome Effects of Serious Gaming as an Adjunct to Treatment for Children With Attention-Deficit/Hyperactivity Disorder: A Randomized Controlled Trial [5] | Low | Some concerns | Some concerns | Some concerns | Low | Some concerns |  |
| Improving Executive Functioning in Children with ADHD: Training Multiple Executive Functions within the Context of a Computer Game. A Randomized Double-Blind Placebo Controlled Trial [6] | Low | Low | Low | Low | Low | Low |  |
| The Effects of Exergaming on Attention in Children With Attention Deficit/Hyperactivity Disorder: Randomized Controlled Trial. [7] | Low | Some concerns | Low | Low | Some concerns | Some concerns |  |
| TARLAN: A Simulation Game to Improve Social Problem-Solving Skills of ADHD Children [8] | Low | Some concerns | Low | Low | Some concerns | Some concerns |  |
|  |  |  |  |  |  |  | *Bias ratings: Low risk of bias, High risk of bias, Some concerns |
| **Table S2.** Quantitative research. | | | | | | | |
| Cochrane Risk of Bias tool for Non-randomized Studies of Interventions (ROBINS-I) | Risk of bias due to confounding | Risk of bias in classification of interventions | Risk of bias in selection of participants into the study (or into the analysis) | Risk of bias due to deviations from intended interventions | Risk of bias due to missing data | Risk of bias arising from measurement of the outcome | Risk of bias in selection of the rep |
| Development of virtual reality rehabilitation games for children with attention-deficit hyperactivity disorder [9] | Serious | Low | Serious | Low | Low | Moderate | Low |
| Multisensory Virtual Game with Use of the Device Leap Motion to Improve the Lack of Attention in Children of 7–12 Years with ADHD [10] | Serious | Low | Serious | Low | Low | Moderate | Low |
| Novel Interactive Eye-Tracking Game for Training Attention in Children With Attention-Deficit/Hyperactivity Disorder.[11] | Serious | Low | Moderate | Low | Low | Moderate | Low |
| Development of Serious Games for Neurorehabilitation of Children with ADHD through Neurofeedback [12] | Serious | Low | Moderate | Low | Low | Moderate | Low |
| Educational games based on distributed and tangible user interfaces to stimulate cognitive abilities in children with ADHD [13] | Serious | Low | Moderate | Low | Low | Moderate | Low |
| Exploring Learning in Near-Field Communication-Based Serious Games in Children Diagnosed with ADHD [14] | Moderate | Low | Moderate | Low | Low | Moderate | Low |
| Influence of a BCI neurofeedback videogame in children with ADHD: Quantifying the brain activity through an EEG signal processing dedicated toolbox [15] | Serious | Low | Moderate | Low | Low | Moderate | Low |
| NEUROBOT: A psycho-edutainment tool to perform neurofeedback training in children with ADHD [16] | Serious | Low | Moderate | Low | Low | Moderate | Low |
| Neurofeedback Based Attention Training for Children with ADHD [17] | Serious | Low | Moderate | Low | Low | Moderate | Low |
| Quantifying Brain Activity State: EEG analysis of Background Music in A Serious Game on Attention of Children [18] | Serious | Low | Moderate | Low | Low | Moderate | Low |
| Eye-Contact Game Using Mixed Reality for the Treatment of Children With ADHD [19] | Serious | Low | Moderate | Low | Low | Moderate | Low |
| Effectiveness of a digital therapeutic as adjunct to treatment with medication in pediatric ADHD [20] | Serious | Low | Moderate | Low | Low | Moderate | Low |
| Antonyms: A Computer Game to Improve Inhibitory Control of Impulsivity in Children with ADHD [21] | Moderate | Low | Moderate | Low | Low | Moderate | Low |
| Development and Validation of a Gamified Videogame for Math Learning in Attention Deficit Hyperactivity Disorder Children (ADHD) [22] | Moderate | Low | Moderate | Low | Low | Moderate | Low |
| Dilud: A Mobile Application to Reinforce Rote Learning in Elementary School Children with Attention Deficit Hyperactivity Disorder [23] | Serious | Low | Moderate | Low | Low | Moderate | Low |
| Empowering children with ADHD learning disabilities with the Kinems Kinect learning games [24] | Serious | Low | Moderate | Low | Low | Moderate | Low |
| PigScape: An embodied video game for cognitive peer-Training of impulse and behavior control in children with ADHD [25] | Serious | Low | Moderate | Low | Low | Moderate | Low |
| Serious Games and Their Effect Improving Attention in Students with Learning Disabilities [26] | Moderate | Low | Low | Low | Low | Moderate | Low |
| BRAVO: A Gaming Environment for the Treatment of ADHD [27] | Moderate | Low | Low | Low | Low | Moderate | Low |
| Adjuvant Therapy for Attention in Children with ADHD Using Game-Type Digital Therapy [28] | Moderate | Low | Low | Low | Low | Moderate | Low |
| **Table S3.** Qualitative research. | | | | | | | |
| CASP (Critical Appraisal Skills Programme) Checklists | Was there a clear statement of the aims of the research? | Is a qualitative methodology appropriate? | Was the research design appropriate to address the aims of the research? | Was the recruitment strategy appropriate to the aims of the research? | Was the data collected in a way that addressed the research issue? | Has the relationship between researcher and participants been adequately considered? | Have ethical issues been taken into consideration? |
| A 3D Rhythm-based Serious Game for Collaboration Improvement of Children with ADHD [29] | Yes | Yes | Yes | Can't Tell | Yes | No | Can't Tell |
| Designing MIND PRO Working Memory Game and evaluating its effectiveness on working memory in ADHD children [30] | Yes | Can't Tell | Yes | No | Yes | Can't Tell | Can't Tell |
| Developing an edutainment game, taboo!, for children with ADHD based on socially aware design and VCIA model [31] | Yes | Yes | Yes | Yes | Yes | Can't Tell | Yes |
| Developing and feasibility testing of the Indonesian computer-based game prototype for children with attention deficit/hyperactivity disorder [32] | Yes | Yes | Yes | Yes | Yes | Can't Tell | Yes |
| Towards the improvement of ADHD children through augmented reality serious games: Preliminary results [33] | Yes | Can't Tell | Yes | Yes | Yes | Can't Tell | Yes |
| DIVIDI2: Reinforcing Divided Attention in Children with AD/HD Through a Mobile Application [34] | Yes | Can't Tell | Yes | Yes | Yes | Can't Tell | Yes |
| KAPEAN: Understanding Affective States of Children with ADHD [35] | Yes | Yes | Yes | Yes | Yes | Can't Tell | Yes |

### **References**

1. Rodrigo-Yanguas M, Martin-Moratinos M, Menendez-Garcia A, Gonzalez-Tardon C, Royuela A, Blasco-Fontecilla H. A virtual reality game (The Secret Trail of Moon) for treating attention-deficit/hyperactivity disorder: development and usability study. JMIR Serious Games 2021; 9(3):e26824
2. Kanellos T, Doulgerakis A, Georgiou E, Bessa M, Thomopoulos S, Vatakis A. User experience evaluation of the REEFOCUS ADHD management gaming system. 2019. Presented at: 4th International Conference on Smart and Sustainable Technologies (SpliTech); 2019 June 18-21; Split, Croatia. p. 1-6
3. Schena A, Garotti R, D'Alise D, Giugliano S, Polizzi M, Trabucco V, Riccio MP, Bravaccio C. IAmHero: preliminary findings of an experimental study to evaluate the statistical significance of an intervention for ADHD conducted through the use of serious games in virtual reality. Int J Environ Res Public Health 2023; 20(4):0
4. Weerdmeester J, Cima M, Granic I, Hashemian Y, Gotsis M. A feasibility study on the effectiveness of a full-body videogame intervention for decreasing attention deficit hyperactivity disorder symptoms. Games Health J 2016; 5(4):258-269
5. Bul KCM, Kato PM, Van der Oord S, Danckaerts M, Vreeke LJ, Willems A, van Oers HJJ, Van Den Heuvel R, Birnie D, Van Amelsvoort TAMJ, Franken IHA, Maras A. Behavioral outcome effects of serious gaming as an adjunct to treatment for children with attention-deficit/hyperactivity disorder: A randomized controlled trial. J Med Internet Res 2016; 18(2):e26
6. Dovis S, Van der Oord S, Wiers RW, Prins PJM. Improving executive functioning in children with ADHD: training multiple executive functions within the context of a computer game. a randomized double-blind placebo controlled trial. PLoS One 2015; 10(4):e0121651
7. Ji H, Wu S, Won J, Weng S, Lee S, Seo S, Park JJ. The effects of exergaming on attention in children with attention deficit/hyperactivity disorder: randomized controlled trial. JMIR Serious Games 2023; 11:e40438
8. Ahmadi A, Mitrovic A, Najmi B, Rucklidge J. TARLAN: a simulation game to improve social problem-solving skills of ADHD children. In: Artificial Intelligence in Education. Cham: Springer International Publishing; 2015. 328-337
9. Ou Y, Wang Y, Chang H, Yen S, Zheng Y, Lee B. Development of virtual reality rehabilitation games for children with attention-deficit hyperactivity disorder. J Ambient Intell Human Comput 2020; 11(11):5713-5720
10. Capelo DC, Sánchez ME, Hurtado JS, Chicaiza DB. Multisensory virtual game with use of the device leap motion to improve the lack of attention in children of 7–12 years with ADHD. 2018. Presented at: Proceedings of the International Conference on Information Technology & Systems (ICITS 2018); 2018 January 10-12; Ecuador. p. 897-906
11. García-Baos A, D'Amelio T, Oliveira I, Collins P, Echevarria C, Zapata LP, Liddle E, Supèr H. Novel interactive eye-tracking game for training attention in children with attention-deficit/hyperactivity disorder. Prim Care Companion CNS Disord 2019; 21(4):0
12. Machado FSV, Casagrande WD, Frizera A, Rocha FEM. Development of serious games for neurorehabilitation of children with attention-deficit/hyperactivity disorder through neurofeedback. 2019. Presented at: 18th Brazilian Symposium on Computer Games and Digital Entertainment (SBGames); 2019 October; Rio de Janeiro, Brazil. p. 91-97
13. de la Guía E, Lozano MD, Penichet VMR. Educational games based on distributed and tangible user interfaces to stimulate cognitive abilities in children with ADHD. Brit J Educational Tech 2014; 46(3):664-678
14. Avila-Pesantez D, Santillán GS, Padilla N, Miriam AL, Arellano-Aucancela A. Exploring learning in near-field communication-based serious games in children diagnosed with ADHD. 2021. Presented at: Advances in Emerging Trends and Technologies; 2021 May 29-31; Quito, Ecuador. p. 314-324
15. Blandon DZ, Munoz JE, Lopez DS, Gallo OH. Influence of a BCI neurofeedback videogame in children with ADHD. Quantifying the brain activity through an EEG signal processing dedicated toolbox. 2016. Presented at: IEEE 11th Colombian Computing Conference (CCC); 2016 September 27-30; Popayan, Colombia. p. 1-8
16. Vita S, Mennitto A. Neurobot: a psycho-edutainment tool to perform neurofeedback training in children with ADHD. 2019. Presented at: CEUR Workshop Proceedings; 2019 November 25-26; Naples; Italy.
17. Chen CL, Tang YW, Zhang NQ, Shin J. Neurofeedback based attention training for children with ADHD. 2017. Presented at: IEEE 8th International Conference on Awareness Science and Technology (iCAST); 2017 November 08-10; Taichung, Taiwan. p. 93-97
18. Soysal ÖM, Kiran F, Chen J. Quantifying brain activity state: EEG analysis of background music in a serious game on attention of children. 2020. Presented at: 4th International Symposium on Multidisciplinary Studies and Innovative Technologies (ISMSIT); 2020 October 22-24; Istanbul, Turkey. p. 1-7
19. Kim S, Ryu J, Choi Y, Kang Y, Li H, Kim K. Eye-contact game using mixed reality for the treatment of children with attention deficit hyperactivity disorder. IEEE Access 2020; 8:45996-46006
20. Kollins SH, Childress A, Heusser AC, Lutz J. Effectiveness of a digital therapeutic as adjunct to treatment with medication in pediatric ADHD. NPJ Digit Med 2021; 4(1):58
21. Crepaldi M, Colombo V, Mottura S, Baldassini D, Sacco M, Cancer A, Antonietti A. Antonyms: A computer game to improve inhibitory control of impulsivity in children with attention deficit/hyperactivity disorder (ADHD). Information 2020; 11(4):230
22. Castro R, Huamanchahua D. Development and validation of a gamified videogame for math learning in attention deficit hyperactivity disorder children (ADHD). 2021. Presented at: CEUR Workshop Proceedings; 2021 November 16-18; Chiclayo, Peru. p. 17-25
23. Celis G, Casas M, Mauricio D, Santisteban J. Dilud: A mobile application to reinforce rote learning in elementary school children with attention deficit hyperactivity disorder. Int. J. Interact. Mob. Technol 2023; 17(06):62-80
24. Retalis S, Korpa T, Skaloumpakas C, Boloudakis M, Kourakli M, Altanis G, Siameri F, Papadopoulou P, Lytra F, Pervanidou P. Empowering children with ADHD learning disabilities with the kinems kinect learning games. 2014. Presented at: 8th European Conference on Games Based Learning; 2014 October 9-10; Berlin, Germany. p. 28-39
25. Gizatdinova Y, Remizova V, Sand A, Sharma S, Rantanen K, Helminen T, Kylliäinen A. PigScape: An embodied video game for cognitive peer-training of impulse and behavior control in children with ADHD. 2022. Presented at: ASSETS '22: Proceedings of the 24th International ACM SIGACCESS Conference on Computers and Accessibility; 2022 October 23 - 26; Athens Greece. p. 1-4
26. García-Redondo P, García T, Areces D, Núñez JC, Rodríguez C. Serious games and their effect improving attention in students with learning disabilities. Int J Environ Res Public Health 2019; 16(14):2480
27. Barba MC, Covino A, De LV, DePaolis LT, D'Errico G, Di B. BRAVO: a gaming environment for the treatment of ADHD. In: Augmented Reality, Virtual Reality, and Computer Graphics. Cham: Springer International Publishing; 2019.
28. Kim S, Lee H, Lee H, Kim G, Song J. Adjuvant therapy for attention in children with ADHD using game-type digital therapy. Int J Environ Res Public Health 2022; 19(22):14982
29. Giannaraki M, Moumoutzis N, Papatzanis Y, Kourkoutas E, Mania K. A 3D rhythm-based serious game for collaboration improvement of children with attention deficit hyperactivity disorder (ADHD). 2021. Presented at: IEEE Global Engineering Education Conference (EDUCON); 2021 April 21-23; Vienna, Austria. p. 1217-1225
30. Aghdam KS, Alavi MH. Designing MIND PRO working memory game and evaluating its effectiveness on working memory in ADHD children. 2019. Presented at: International Serious Games Symposium (ISGS); 2019 December 26; Tehran, Iran. p. 124-128
31. Batista BG, Rodrigues AFD, Miranda DM, Ishitani L, Nobre CN. Developing an edutainment game, taboo!, for children with ADHD based on socially aware design and VCIA model. 2022. Presented at: IHC '22: Proceedings of the 21st Brazilian Symposium on Human Factors in Computing Systems; 2022 October 17 - 21; Diamantina Brazil. p. 1-11
32. Wiguna T, Ismail RI, Kaligis F, Minayati K, Murtani BJ, Wigantara NA, Pradana K, Bahana R, Dirgantoro BP, Nugroho E. Developing and feasibility testing of the Indonesian computer-based game prototype for children with attention deficit/hyperactivity disorder. Heliyon 2021; 7(7):e07571
33. Avila-Pesantez D, Rivera LA, Vaca-Cardenas L, Aguayo S, Zuñiga L. Towards the improvement of ADHD children through augmented reality serious games: preliminary results. 2018. Presented at: IEEE Global Engineering Education Conference (EDUCON); 2018 April 17-20; Santa Cruz de Tenerife, Spain. p. 843-848
34. Jácome V ID, Páez O JS, Cóllazos O CA, Fardoun HM. DIVIDI2: reinforcing divided attention in children with AD/HD through a mobile application. 2019. Presented at: REHAB '19: Proceedings of the 5th Workshop on ICTs for improving Patients Rehabilitation Research Techniques; 2019 September 11 - 13; Popayan Columbia. p. 106-110
35. Fernando M, Claudia B, Nimrod G, Juan G. KAPEAN: understanding affective states of children with ADHD. Journal of Educational Technology & Society 2016; 19(2):18-28
